# Inverse Design of Nanoparticulate Materials

**DOI:** 10.1002/adma.75110

**Published:** 2026-09-25

**Authors:** Nabi Etienne Traoré, Annika Mauch, Michelle Berthold, Lukas Pflug, Wolfgang Peukert, Nicolas Vogel

**Affiliations:** ^1^ Institute of Interfaces and Particle Technology (IPT) Friedrich‐Alexander‐Universität Erlangen‐Nürnberg Erlangen Germany; ^2^ Erlangen Center for Functional Particle Systems (FPS) Friedrich‐Alexander‐Universität Erlangen‐Nürnberg Erlangen Germany; ^3^ FAU Competence Center Scientific Computing (FAU CSC) Friedrich‐Alexander‐Universität Erlangen‐Nürnberg Erlangen Germany

**Keywords:** inverse design, modeling, nanomaterials, nanoparticles, optimization, property–process relationship

## Abstract

The unique size‐ and shape‐dependent properties of nanomaterials offer a rich parameter space for tailoring functionalities and applications. Recently, inverse design of such nanoparticulate systems has provided a paradigm shift from empirical trial‐and‐error approaches toward predictive, model‐driven design strategies to achieve desired functionalities. This perspective presents a practical framework for applying inverse design to nanoparticulate materials. We distinguish between two general modeling strategies: knowledge‐based design, grounded in a detailed understanding of the underlying physics and chemistry, and data‐based design, based on experimental or simulated input–output datasets. Hybrid models bridge these two strategies. Each strategy is further structured into three levels of optimization: (i) process optimization via process functions connecting synthetic parameters with resulting particle properties; (ii) structure optimization via property functions connecting particle properties with macroscopic properties; and (iii) full inverse design via combined property–process relationships. In a tutorial style, we introduce practical steps for model development, calibration, and implementation and discuss design rules to guide the choice of modeling strategy. This perspective thus aims to facilitate the broad adoption of inverse design for nanoparticulate systems, laying the foundation for the development of rigorously optimized, application‐specific materials with ideal properties tailored to a given application.

## Graphical abstract

Inverse design shifts nanomaterial development from empirical trial‐and‐error to predictive model‐driven strategies. It can rely on knowledge‐based, data‐based, or hybrid process and property functions. This perspective article provides a practical framework for applying inverse design based on instructive examples. It discusses which modeling approach is best suited for different scenarios and what is required for successful implementation.

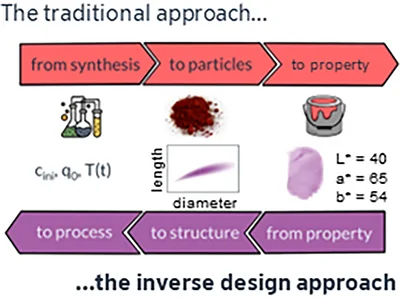

## 1 Introduction

Nanoparticles (NPs), typically defined as particles ranging from 1 to 100 nm in size [1], exhibit pronounced size‐dependent properties that differ fundamentally from those of their bulk counterparts. These include optical [2, 3, 4, 5, 6], electrical [7, 8, 9], magnetic [10, 11, 12, 13], and catalytic [14, 15] properties, which have led to their widespread application across fields such as nanophotonics [16, 17, 18, 19, 20], microelectronics [21, 22], biosensing [23, 24], catalysis [25], or theranostics [26, 27, 28]. Rationally designing such nanoparticulate products requires precise control over their disperse properties (e.g., size, shape, composition, ligand shell, etc.), a challenge that has been the focus of intense research for many years [29, 30, 31, 32, 33, 34, 35, 36, 37].

Traditionally, tailoring these parameters for a specific application has relied on systematically varying synthesis conditions and observing the resulting macroscopic behavior. However, due to the increasing structural complexity of nanoparticulate systems and the nonlinear relationships between synthesis parameters, particle structure, and resulting (macroscopic) properties [38, 39], empirical approaches increasingly reach their limits. While advanced design‐of‐experiments strategies help explore parameter spaces more systematically [40, 41, 42, 43], several fundamental questions about nanomaterial design remain:

- *Was the optimal structure for the desired property synthesized or even identified?* Even if a refined synthesis yields a nanoparticle structure with properties close to the target, it remains unclear whether an alternative structure might perform even better for the intended application.
- *How robust is the identified structure with respect to the desired property?* An identified nanoparticle structure may achieve the targeted property, but for practical use it is crucial to know whether this property is robust against inevitable variations in the NPs’ disperse properties during synthesis. Robust solutions are generally preferred.
- *Does a particle structure providing the targeted property even exist?* Exploring parameter spaces within known boundaries may lead to suitable structures, but being able to determine in advance whether the desired property is achievable within a given material system or synthesis route may save significant time and resources.
- *Does the synthesis route allow for applications beyond the initial scope?* While optimization requires that a target property (e.g., a resonance position) is defined in advance, the broadness of the accessible property space ultimately determines which applications become feasible. A rigorous search for appropriate parameters may extend the accessible property range, thereby uncovering opportunities and potentials that would otherwise remain overlooked.

Inverse design is a conceptually different approach toward the design of (nano)materials with the potential to overcome these challenges by inverting the process chain. It starts with mathematical modeling and optimization approaches that are employed to identify optimal particle structure and process parameters for a desired property [44]. Once this ideal structure or process is identified, it serves as a blueprint that can be experimentally realized without having to screen a large parameter space. Inverse design has a long history in materials science [45, 46, 47, 48, 49, 50, 51, 52, 53], with successful applications in fields ranging from crystalline materials [46, 52], to polymers [47], and metals [49, 50], as well as the (bio)chemical field [54, 55, 56, 57, 58, 59, 60, 61], including ionic liquids [54], chemical compounds [56], drugs [60, 61, 62], and fragrance design [55]. However, transferring these concepts to the design of nanoparticulate systems has only recently gained traction, likely driven in part by advances in artificial intelligence [63, 64, 65] and the broader adoption of laboratory automation technologies [66, 67, 68, 69, 70]. Nanoparticulate materials in particular are defined by distributions in the NPs’ disperse properties (i.e., their structural parameters such as size, shape, composition, and their distributions around a mean value) [71, 72]. The fact that no two particles are identical thus adds an additional layer of complexity to the design problem, as the distribution of size, shape, and other structural parameters consequently leads to a distribution of properties.

This perspective aims to provide a practical guide for the application of inverse design to the targeted synthesis of nanoparticulate materials. We adopt a tutorial‐style approach with the aim to make the concept accessible to a broad readership. We select examples focusing on different properties, including optical, magnetic, or catalytic nanomaterials to showcase the utility of inverse design across different application scenarios. For an in‐depth introduction to the implementation of inverse design, the discussion of the underlying framework, and the comparison of different approaches, we then pick the example of optical properties as an instructive application scenario. Optical properties are instructive to understand the concepts underpinning inverse design for two reasons. First, physical structure–property relations connecting the size and shape of particles to their optical properties are readily available via Maxwell's equations for sizes above the limit of quantum confinement [73, 74, 75]. Second, optical properties are very sensitive to the structural properties of particulate systems [76], providing a complex design space with the potential to truly optimize a structure to a target property. Notably, this sensitivity extends to distributions in these structural parameters. Since real particle ensembles inevitably exhibit dispersity in size, shape, and composition, even moderate variations around a mean structure can substantially alter the ensemble‐averaged optical response. How such distributions are accounted for is therefore an important consideration in any inverse design approach involving nanoparticulate systems. In practice, optical properties of nanomaterials are of direct relevance for aesthetic applications as efficient colorants [77, 78, 79], in display technology [80, 81], or as nanophotonic devices [16, 17, 82]. Their importance, however, extends to a range of additional applications that are associated with strong light–matter interactions enabled by plasmonic particles, such as efficient biosensing [23, 83, 84, 85, 86], photocatalysis [87, 88], antimicrobial properties [89, 90, 91], or photothermal heating in cancer treatment [92, 93, 94, 95, 96, 97]. Numerous NP systems with tailored optical properties originating from different light–matter interactions have been developed and reported in the literature.

Plasmonic NPs exhibit size‐, shape‐, and composition‐dependent optical resonances as a result of their localized surface plasmon resonances (LSPR). These resonances arise from the interaction of light with their electron plasma, resulting in collective oscillations due to the absorption of specific wavelengths from the incident white light [98, 99, 100]. The resonance structure can be further tuned via plasmon hybridization by bringing individual particles in close proximity [16, 101, 102], or by arraying particles into defined lattices, which can lead to the occurrence of surface lattice resonances [103, 104, 105], and may even exhibit chiral properties [106, 107].

Figure 1A exemplarily shows the ability to tailor plasmonic coloration by changing the dimension of a patch of plasmonic material formed on a dielectric core particle (Figure 1A‐i). Increasing coverages of the core particles lead to a shift of the LSPR position to higher wavelengths [108, 109]. Similarly, changes in the composition, for example, by increasing the molar gold content of bimetallic silver–gold (AgAu) alloy NPs shift the LSPR position to higher wavelengths (Figure 1A‐ii) [110, 111].

**FIGURE 1:**
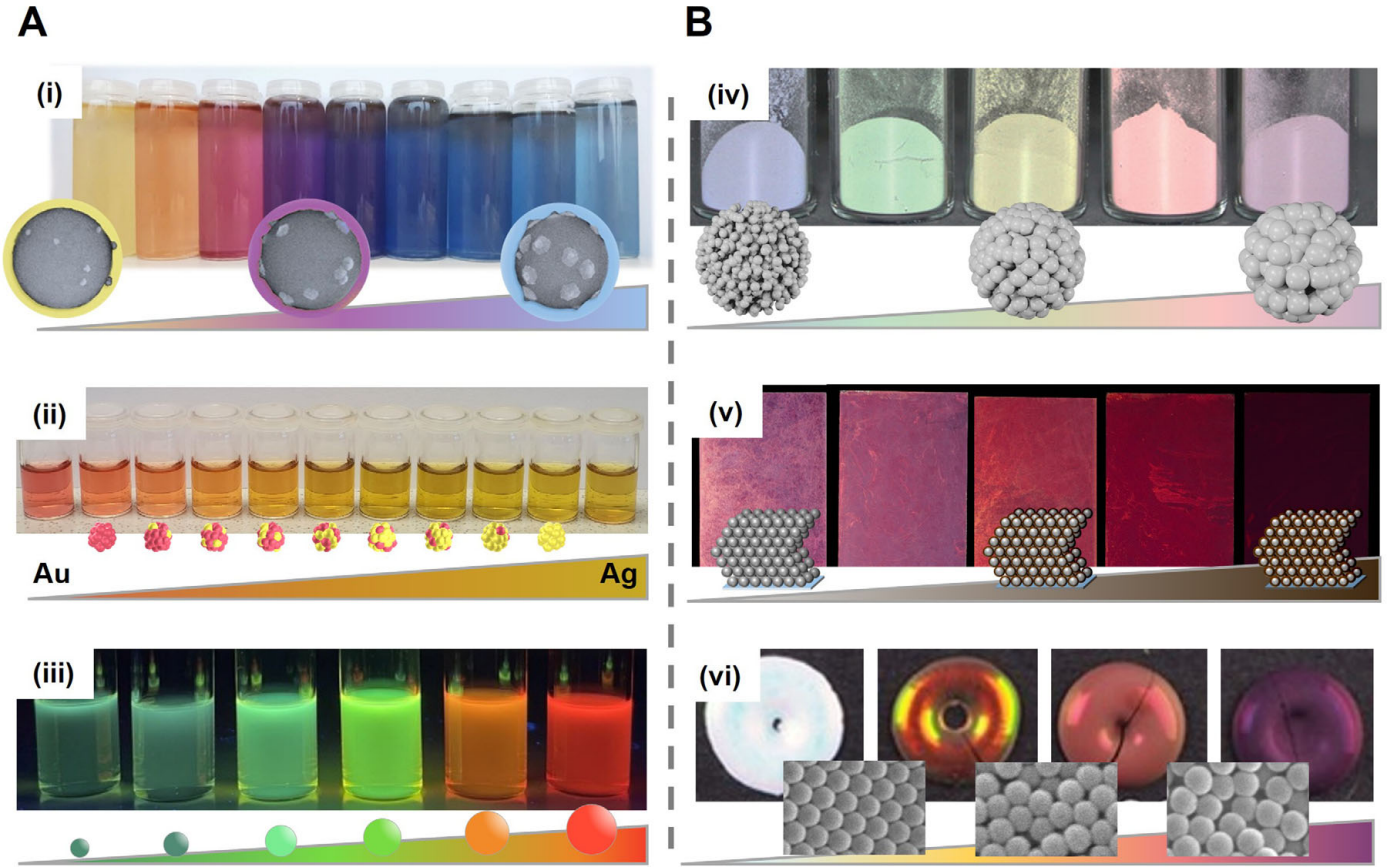
Optical properties of nanoparticulate materials. (A) Optical properties of nanoparticle dispersions as a function of nanoparticle shape, composition, and size. (i) Aqueous dispersion of silver@silica patchy nanoparticles with increasing patch size from left to right. Reprinted (adapted) with permission from Ref. [108]. Copyright 2023 American Chemical Society. (ii) Aqueous dispersions of silver–gold alloy nanoparticles with increasing molar silver content from left to right. Reproduced from Ref [110], DOI: 10.1039/D2NA00793B, under the terms of the CC BY 3.0 license (https://creativecommons.org/licenses/by/3.0/). Graphical representations of alloy nanoparticles were added. (iii) Dispersions of indium‐phosphide/zinc‐sulfide core–shell quantum dots with increasing core diameters from left to right. (B) Optical properties of photonic nanoparticulate assemblies as a function of primary particle size, absorber content, and particle order. (iv) Silica supraparticle powders with increasing primary particle size from left to right. Reproduced from Ref. [142], DOI: 10.1002/smll.202203068, under the terms of the CC BY 4.0 license (https://creativecommons.org/licenses/by/4.0/). Graphical representations of supraparticles were added. (v) Macroscopic photonic crystals on microscopy slides formed from polystyrene (PS) @polydopamine (PDA) core–shell particles with increasing light‐absorbing PDA shell thicknesses from left to right. Reproduced from Ref. [151], DOI: 10.1002/adom.202403279, under the terms of the CC BY 4.0 license (https://creativecommons.org/licenses/by/4.0/). Panels were cropped and rearranged. (vi) Pellets of PS@PDA core–shell particles with increasing light‐absorbing PDA shell thicknesses from left to right. The resulting increase in particle diameter and surface roughness induces disorder into the pellet, leading to an increasingly matte appearance of the pellets. Reproduced from Ref. [124] DOI: 10.1038/srep33984, under the terms of the CC BY 4.0 license (https://creativecommons.org/licenses/by/4.0/). Panels were cropped and rearranged.

Quantum dots (QDs), semiconductor particles with a diameter typically in the range of 2–10 nm [112, 113], exhibit broad absorption and narrow emission spectra that are sensitive to their size as a result of quantum confinement. Their color thus arises from the size‐dependent bandgap energy [114], which causes a blueshift of the emitted color with decreasing QD size [115]. Exemplarily, Figure 1A‐iii demonstrates this size‐dependent luminescence for InP/ZnS core–shell QDs, which, due to their low toxicity and broad color tunability, receive increasing interest as a possible alternative to heavy metal‐based QDs [116, 117].

Lastly, optically‐active nanomaterials can also be formed by the assembly of individual particles into defined structures, termed colloidal photonic crystals [118, 119, 120, 121, 122, 123]. Structural color arises from the constructive interference of the light scattered at these arranged primary particles. Photonic crystals possess a regular, periodic nanostructure composed of two or more materials with different refractive indices [74, 75, 122]. When light is incident on the crystal, it interacts with the material interfaces, where reflection, scattering, and diffraction occur. The partially reflected waves then interfere and certain wavelengths are enhanced by constructive interference, while others are suppressed by destructive interference. Because the condition for constructive interference changes with the angle of the incident light, ordered photonic crystals typically produce angle‐dependent colors [120, 122, 124]. Colloidal assemblies with only short‐range positional order, commonly referred to as photonic glasses, produce angle‐independent structural colors [125]. In such systems, color arises not from Bragg diffraction but from coherent scattering at a characteristic length scale set by the mean nearest‐neighbor particle distance [125, 126]. The resulting optical response is more isotropic, making photonic glasses particularly attractive for pigment applications.

The physical phenomena underlying structural color in colloidal assemblies, that is, scattering and absorption, are based on topics studied by the optics and photonics communities, often under different terminology. At the single‐particle level, the scattering and absorption cross‐sections of the primary particles are governed by the properties of the primary particles, while the collective optical response of the assembly emerges from scattering, interference, and photonic bandgap effects [75, 126]. Beyond colloidal self‐assembly, the same optical physics governs color generation in nanostructures produced by top‐down fabrication methods. For example, in surface nanostructures, color arises from the (resonant) interactions between light and the geometry of the structure [127, 128]. More broadly, the same optimization logic that governs the inverse design of structural color extends to other optical targets rooted in the same scattering physics. A prominent example is the maximization of broadband diffuse reflectance, creating a “perfect white”, where the goal is to maximize incoherent multiple scattering across the visible spectrum [129, 130, 131, 132, 133]. Similarly, the haze factor, which is defined as the fraction of transmitted light that is scattered, is a target optical property of direct relevance for functional coatings, solar cell light management layers, and transparent electrodes, and can be optimized by tailoring the structure of particulate coatings [134].

In nature, structural color is often employed to create the vibrant coloration of butterflies, beetles, or bird feathers [135, 136, 137, 138, 139]. The optical appearance of such photonic structures can synthetically be recreated by colloidal photonic crystals and the color controlled via the used primary particles (Figure 1B). Increasing the size of the primary particles within the assembled material changes the conditions for constructive interference and causes a redshift of the observable structural color (Figure 1B‐iv) [140, 141, 142]. Changing the amount of absorbing material incorporated within colloidal photonic structures alters the saturation and chroma of the resultant structural color [143, 144, 145, 146, 147, 148, 149, 150]. With the addition of absorbing primary particles, the color saturation first increases as incoherently scattered light at structural defects is removed, but eventually turns into dark hues with increasing absorber content (Figure 1B‐v). The structure of the self‐assembled material further affects the optical properties: photonic crystals with ordered arrangement of the primary particles produce iridescent structural color with pronounced angle‐dependent appearances, while photonic glasses with short‐range order produce non‐iridescent, angle‐independent structural color (Figure 1B‐vi) [120, 122, 124]. Colloidal photonic crystals can exhibit vibrant structural colors, offering a sustainable and long‐term stable alternative to conventional organic dyes [121, 122, 143].

To illustrate how mathematical models can be used to enable the inverse design of such nanoparticulate materials with tailored optical, magnetic, or catalytic properties, we structure this article around two general modeling strategies: knowledge‐based design, rooted in a detailed understanding of the underlying physics and chemistry of both the formation process and the desired target property, and data‐based design, which is based on experimental or simulated datasets to train surrogate models. Each strategy is further subdivided into three types of optimization approaches, i.e., (i) process optimization via process functions, connecting synthetic parameters with resulting particle properties, (ii) structure optimization via property functions, connecting particle properties with the resultant macroscopic properties, and (iii) full inverse design using combined property–process relationships. For each category, we discuss representative case studies and outline the underlying methodologies employed for model development and optimization via inverse design. By clearly defining the types of inverse design models and their practical implementation, this perspective seeks to encourage broader adoption of inverse design strategies in nanoparticulate research, well beyond the use of color as a demonstrator property.

## 2 Conceptual Frameworks

Figure 2A contrasts the conventional method for the synthesis of nanoparticulate materials with the predictive, inverse design strategy to yield optimized properties, using optical properties as an example for the target property. In the traditional approach, particles with varying disperse properties (e.g., size, shape, and composition) are synthesized using different process conditions (e.g., initial precursor concentrations *c*_ini_, initial particle size distributions (PSDs) *q*_0_, temperatures at specific times throughout the synthesis *T*(*t*), etc.) to screen a parameter space, yielding particle systems with certain structural properties. These particles are then characterized to investigate their macroscopic properties, such as the observable color. The accurate comparison of the performance of the synthesized particle system, that is, how close the obtained properties are to a predefined target, requires quantification of color properties. This quantification is implemented by measuring their spectral properties, typically using extinction or transmission spectroscopy. The obtained spectra are then translated into color coordinates, using the CIELAB color space. The CIELAB color space (Commission Internationale de l'Éclairage—International Commission on Illumination) is a color system that reflects how humans perceive color. It is therefore used to uniquely identify the color of the particle system as perceived by an observer under standardized illumination conditions using the three parameters L*, a*, and b* [151, 152, 153]. L* represents lightness, with higher values corresponding to lighter (whiter) colors and lower values to darker (blacker) tones. The a* axis spans from green (negative values) to red (positive values), while the b* axis ranges from blue (negative values) to yellow (positive values) [154]. The distance of these color coordinates to the desired target thus serves as a metric to characterize how “good” a given particle structure matches the target hue. To approach a specific predefined color target, process parameters are then iteratively adjusted until a particle system with optical properties that are close to the desired target is found. Note that while this particular quantification is specific to optical properties, the general procedure is not. Catalytic or other properties can equally be quantified by suitable characterization methods and the resulting values compared to a target value.

**FIGURE 2:**
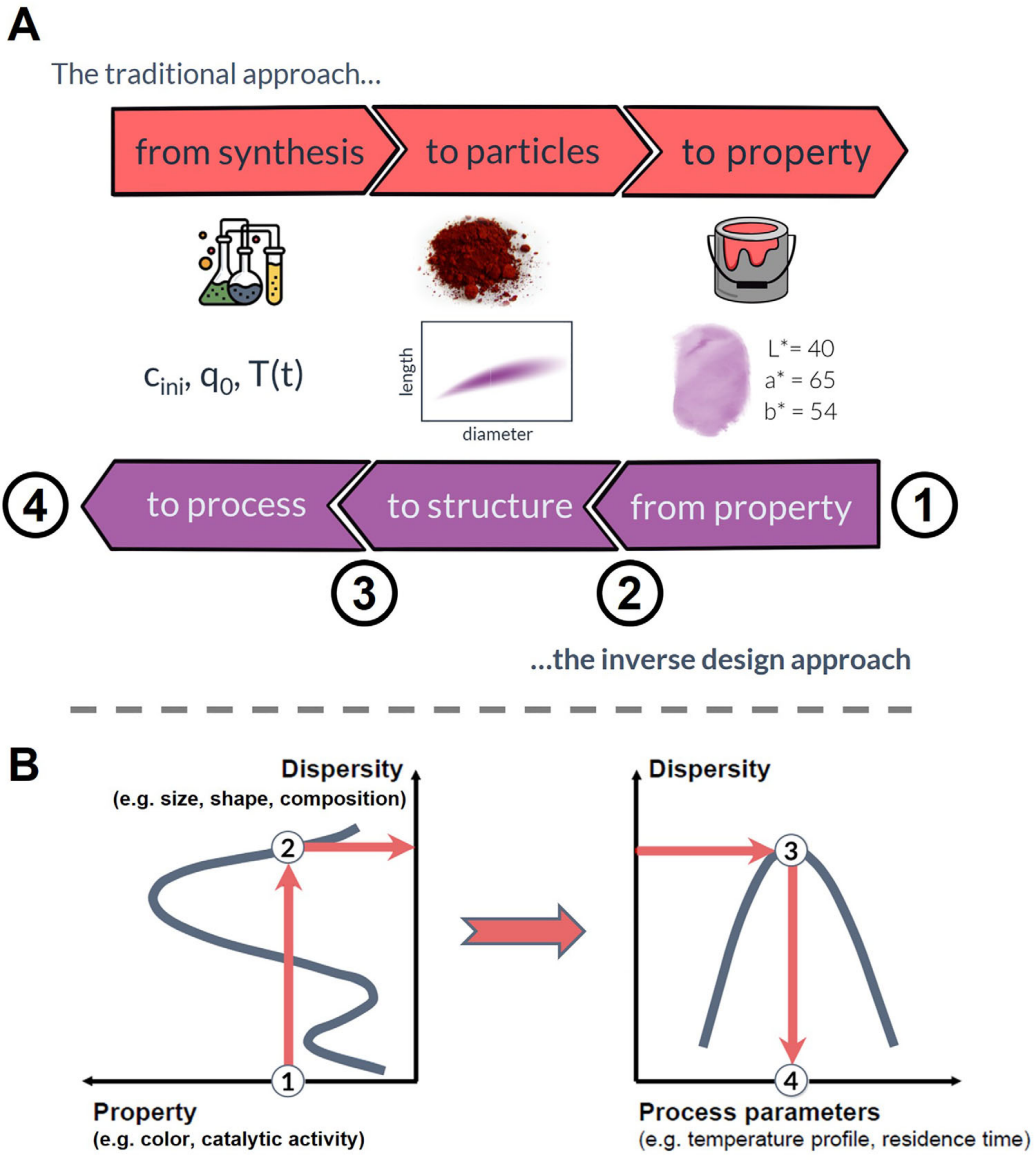
General framework for the inverse design of (nano)particulate materials. (A) Comparison of the traditional design approach, which proceeds from NP synthesis to the characterization of their properties, and the inverse design approach, in which a target property is defined, and models are employed to calculate the required structure (via the property function) and process conditions (via the process function). The numbers 1–4 correspond to the numbers 1–4 in (B). (B) Inverse design of (nano)particulate materials using a property and a process function. A target property of the nanoparticulate system is defined (1) and translated into the required disperse properties (2) using the property function. These disperse properties (3) are subsequently converted into the corresponding process parameters (4) using the process function. The combination of both functions forms a full property–process relationship.

In contrast, the inverse design approach starts with the desired macroscopic property, for example, a specific color target in the CIELAB color space, and then works backward. Based on known structure–property relations, a mathematical optimization framework is established. This framework identifies the optimal particle structure that yields color properties (i.e., color coordinates) which are as close as possible to the desired target properties. Once the optimal dispersity (i.e., particle size, shape, composition, etc.) is identified, a second optimization step identifies ideal process conditions that yield particles with these desired, optimal properties.

Implementing inverse design requires understanding of two key characteristics of the targeted system: the property function, which relates macroscopic properties to the structural identity of the particle system (Figure 2B, left), and the process function, which relates process parameters for NP synthesis to the resulting particle structure (Figure 2B, right). A fully predictive, inverse design workflow combines both functions to form a complete property–structure‐process relationship. Knowledge of both functions thus enables a systematic sequence to identify optimal process conditions for a desired macroscopic property, as illustrated in Figure 2B: (1) defining a target property, (2) calculating the required dispersity via the property function, (3) transferring the required dispersity to the process function, and (4) determining the necessary process parameters to achieve the required dispersity using the process function.

In literature, both functions have been applied individually as well as in combination (full property–process relationship). The isolated use of the process function results in the optimization of a synthesis process, while the isolated use of the property function enables the structural optimization of a nanoparticulate material. Throughout this perspective, we present examples for each of these cases and refer back to Figure 2 to illustrate the corresponding workflows for the inverse design of nanomaterials.

## 3 Inverse Design of Nanoparticulate Materials

Both the property function and the process function can either be derived from a detailed understanding of the underlying physics and chemistry (knowledge‐based approach) or determined from available simulated or experimental data that provide input–output relationships (data‐based approach). Each approach offers distinct advantages and limitations compared to the respective other.

The key characteristic of a knowledge‐based model is that the relationships governing the system are expressed in mechanistic equations whose parameters carry direct physical meaning. When such a model is calibrated against experimental data, the resulting parameter values consequently are physically meaningful quantities, effectively equivalent to measuring those parameters directly. Knowledge‐based process modeling for instance, is enabled by the understanding of complex formation pathways and kinetics underpinning a synthesis process. Conceptually, particle syntheses can be classified into mixing‐controlled and reaction‐controlled systems depending on the rate‐limiting process. Particle formation is governed by the temporal and spatial evolution of supersaturation, determined by the interplay between mixing and reaction rates [155, 156]. In the case of perfect mixing, particle formation can be described by diffusion and reaction rates, with supersaturation dynamics governed exclusively by chemical processes as monomer sources and particle formation processes as monomer sinks. In a knowledge‐based modeling approach, coupling hydrodynamic models with such population balance models enables the prediction of PSDs. Calibrating such models with experimental data helps to validate the assumed reaction steps and often reveals additional mechanistic insights. Once calibrated, these models can be used to estimate experimentally inaccessible parameters, such as reaction rate constants or atom concentration profiles during synthesis [39]. The gained insights of a specific reaction may be transferable to similar formation pathways, providing unifying theories to describe particle syntheses. Thus, the required effort to construct process models for subsequent particle systems tends to decrease, amplifying the utility of the initial modeling framework. For example, a formation model developed for multimaterial indium phosphide (InP) QDs, comprising multiple precursor reactions as well as nucleation and growth steps [112], was readily adapted to bimetallic silver–gold alloy NPs that follow a similar reaction scheme, albeit with additional process steps [39]. Notably, this procedure is not exclusive to optical properties but can be applied to optimize nanoparticle syntheses to any desired property, as long as there is a clear physical connection between structure and property.

Despite these advantages, however, the setup of a knowledge‐based model for a previously unexplored particle system is often associated with high computational and experimental costs [39]. Furthermore, many industrial processes involve complex multicomponent systems, making it challenging, if not impractical, to account for the influence of every component through purely physical descriptions.

In such cases, data‐based modeling offers a practical alternative. Such models do not require prior knowledge of the underlying physical mechanisms, enabling a rapid product optimization directly from empirical data. However, this convenience comes at the cost of interpretability. Data‐based models, especially those based on machine learning algorithms such as neural networks, typically require large datasets to achieve high predictive accuracy [157]. Additionally, their ability to extrapolate beyond the range of training data is often limited, reducing their generalizability [157] and their ability to identify potentially appealing solutions outside the explored parameter spaces. Despite these disadvantages, this approach is particularly useful whenever physical models connecting synthesis and structure, as well as structure and resultant property are not available. Both modeling approaches thus offer advantages that make them suitable for different contexts. In the following sections, we present examples of how each approach has been successfully applied to facilitate the inverse design of nanoparticulate materials.

### 3.1 Knowledge‐Based Optimization

While most inverse design studies of nanoparticulate materials have employed data‐driven methods, several recent works have explored knowledge‐based approaches [39, 112, 151, 152, 158, 159, 160, 161, 162, 163, 164, 165, 166, 167, 168, 169, 170, 171]. Process optimization strategies have been developed for different classes of NPs. For noble metals, these include the prediction of gold NP sizes formed via a citrate‐based synthesis [158], the determination of optimal process parameters for tailoring the size and composition of AgAu alloy NPs based on a stepwise formation mechanism [39], and the inverse design of three‐dimensional noble metal NP morphologies through global optimization using a direct electromagnetic solver [159]. In addition, the size distribution of InP QDs has been optimized via a population balance model, identifying optimal process parameters [112]. Long et al. [163] applied Bayesian optimization to a knowledge‐based population balance model of Ir(0)_n_ nanoparticle formation, extracting rate constants and their uncertainties from experimental size distribution data, enabling process optimization for the synthesis of catalytic NPs. Andalibi et al. [162] similarly developed a population balance model for the precipitation of calcium‐silicate‐hydrate NPs, coupling a speciation solver with global uncertainty and sensitivity analysis to identify which synthesis parameters most sensitively control the specific surface area. Beyond process optimization, structure optimization approaches have been used in a variety of domains comprising optical, catalytic, and magnetic target properties [160, 161, 165, 166, 167, 168, 169, 170, 171]. Amongst others, this includes topology optimization of NPs for spatially localized electric field enhancement [161] and for improving surface‐enhanced Raman scattering (SERS) performance [160], as well as knowledge‐based kinetic Monte Carlo modeling for CO oxidation at RuO_2_(110), directly mapping surface structural parameters to catalytic turnover frequencies [169], and simulations to map the specific loss power as a function of the diameter and thickness of disc‐shaped magnetic NPs, identifying the optimal nanodisc geometry for maximum heating efficiency under biophysical constraints [168]. In each case, the defining feature of the knowledge‐based approach is that the underlying physical or chemical processes are sufficiently well understood to be cast into mathematical equations, so that model parameters remain interpretable as physical quantities. In the following sections, we present selected examples to illustrate the approaches, workflows, and implementations of inverse design in nanoparticulate materials, covering the optimization of NP synthesis processes (Section 3.1.1), nanoparticulate materials structures (Section 3.1.2), and resulting macroscopic properties (Section 3.1.3).

#### 3.1.1 Process Optimization

One instructive example of process optimization using a process function (Figure 2B, right) is the prediction and optimization of the disperse properties of InP QDs [112]. In the example, InP QDs were synthesized using an automated synthesis platform to develop and calibrate a predictive, knowledge‐based model of InP QD formation. The model predicts the disperse properties of the resulting QDs based on selected process parameters, which then enables their subsequent optimization. The development and calibration workflow of the process model is illustrated in Figure 3.

**FIGURE 3:**
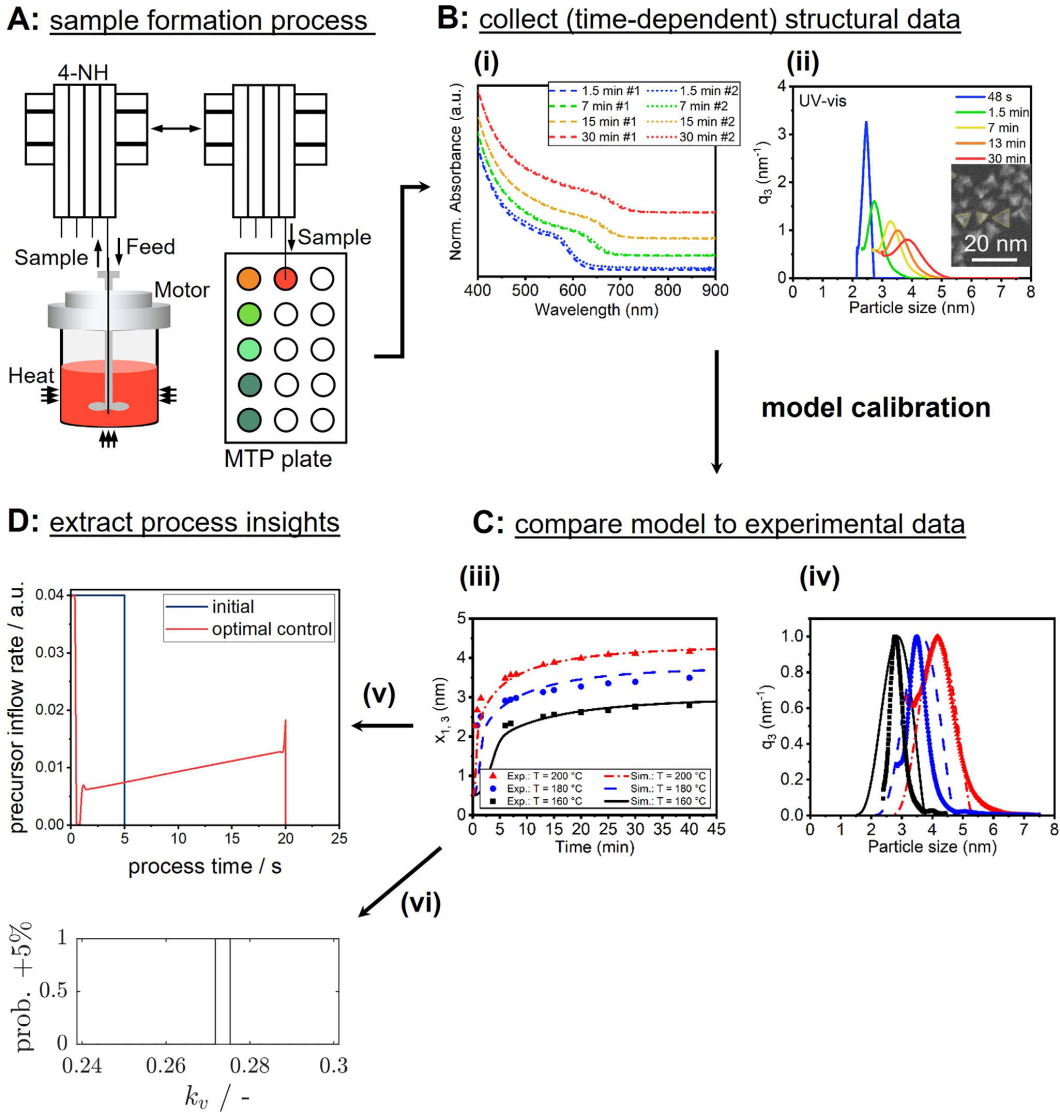
Guide for the inverse design of InP quantum dots (QDs) via process optimization using a knowledge‐based process function, as illustrated in Figure 2. (A) An automated synthesis platform is used to conduct multiple syntheses, during which samples are taken at defined time intervals using its 4‐needle head (4‐NH). Key synthesis parameters, such as temperature and precursor concentrations, are systematically varied. (B) Time‐resolved data on the QDs’ disperse properties are extracted from those samples via the deconvolution of the NPs’ extinction spectra (i) into their particle size distribution (ii). A physical model based on mechanistic correlations is then calibrated using the experimental dataset. (C) Experimental data are compared to predictions of the calibrated model to evaluate its accuracy. Both the mean particle sizes (iii) and the full particle size distributions (iv) are modeled and compared to experimental data. (D) Process insights, including optimal process conditions for targeted NP dispersities (v, Reproduced from Ref [173], DOI: 10.1039/D5RE00333D, under the terms of the CC BY 3.0 license (https://creativecommons.org/licenses/by/3.0/)) and parameters that are experimentally inaccessible (vi, here: the Mersmann constant *k*_**v**_), can be extracted from the calibrated model. If not specified otherwise, reprinted from Chemical Engineering Science, Vol. 281, Z. Wang, N.E. Traoré, T. Schikarski, L.M.S. Stiegler, D. Drobek, B. Apeleo Zubiri, E. Spiecker, J. Walter, W. Peukert, L. Pflug, and D. Segets, “Population balance modeling of InP QDs: Experimentally enabled global optimization to identify unknown material parameters”, 119062, Copyright (2023), with permission from Elsevier [112].

In the first step, a batch synthesis protocol for InP QDs was transferred to a robotic synthesis platform (Figure 3A), which allows for systematic sampling and minimizes sample‐to‐sample variations, known to significantly affect QD synthesis [113], by excluding the human factor (Figure 3B‐i). Fluctuations in experimental data would otherwise hinder the extraction of precise process parameters. Using the robotic platform, a comprehensive dataset of extinction spectra spanning the parameter space of process temperatures, precursor concentrations, and reaction times was thus generated. Because the optical spectra of QDs are known to be size‐dependent and can be deconvoluted into size distributions via their bandgap behavior [172], these spectra were translated into PSDs related to the process conditions used within the synthesis. This approach is particularly useful given the tetrahedral shape of the InP QDs (Figure 3B‐ii, inset), which complicates conventional size characterization due to shape anisotropy. In contrast, the optical deconvolution method was shown to depend only on the volume of the particles, providing a volume‐equivalent diameter distribution suitable for model calibration (see Figure 3B‐ii). This deconvolution directly reflects the sensitivity of optical properties to the full ensemble of particle sizes. Consequently, both model calibration and subsequent process optimization are performed on full distributions.

Building on insights from both experiments and literature, a detailed model of QD formation based on a population balance equation (PBE) coupled with mass balances and precursor conversion reaction kinetics was developed.

First, the network of precursor conversion reactions is mathematically described. During the synthesis, InP monomers are formed through a two‐step mechanism, where first a phosphorus precursor (AP) reacts to form phosphide ions (P^3−^) while accounting for a competing side reaction producing byproducts (BP). In a second step, the phosphide ions then react with indium ions to form InP monomers. The supersaturation of InP monomers then drives both nucleation and growth, whereby the nucleation rate (*N*) is taken from classical nucleation theory (CNT) [156, 174, 175]. These rates feed into a PBE which is coupled with a monomer mass balance via the time‐dependent supersaturation in the system (for details we refer the reader to the original publication) [112].

The final model contains five adjustable parameters, including kinetic constants and CNT‐specific terms, which were then calibrated using the experimental PSD data at different process times and process conditions. To enable this calibration, the exact method of moments (eMoM, [176]) was applied. In practice, this calibration comprises the multivariate global optimization to identify the parameters, which yield the smallest possible deviation between the model and the experimental data. The resulting model represents a process function that maps process parameters to the evolution of QD size distributions.

The model now enables predictions of complete PSDs as a function of process parameters. Figure 3C exemplarily shows a comparison between the model predictions for various process parameters and the corresponding experimental realization. In particular, Figure 3C‐iii shows the mean particle size *x*_1,3_ as a function of process time at different temperatures, while Figure 3C‐iv presents the final PSDs for QDs synthesized at different temperatures. In both diagrams, the solid lines show the predictions obtained from the calibrated process function, while individual symbols represent experimental data. Quantitative agreement between both datasets shows that the process model accurately captures the dominating physicochemical mechanisms, reaction steps, and kinetics underlying the process, and thus enables predicting the full outcome of the synthesis procedure.

Finally, the calibrated process function enables the inverse design of InP QD synthesis by mapping the relationship between synthesis parameters and the resulting disperse properties of the final product. This allows for systematic optimization of the synthesis process by identifying the conditions required to achieve a target structure of the produced NPs, that is, a target size distribution. This strategy is exemplarily shown in Figure 3D‐v. The precursor inflow rate into a batch reactor is optimized to obtain a narrow size distribution at a defined mean particle size.

Beyond enabling the inverse design of InP QDs, the model can also be used to extract parameters, relevant to the system under study, that are difficult or impossible to determine experimentally, because all adjustable parameters employed to calibrate the mechanistic model are grounded in physical meaning (Figure 3D).

In summary, this study illustrates an example for process optimization using a knowledge‐based process function. The approach is versatile, yet it requires substantial initial investment in time and resources to resolve an unknown formation mechanism—an inherent challenge in knowledge‐based modeling. Moreover, since the approach relies on the assumption that a unique solution for the adjustable parameters exists, verifying this uniqueness is essential. When numerous factors influence the process, the optimization task becomes a multidimensional problem, substantially increasing computational costs. These costs can escalate further if the optimum is particularly narrow, necessitating to explore the multidimensional parameter space on a fine grid. In cases where the available data do not allow for a unique solution, additional experimental input is required. Such input should ideally combine dynamic process data (e.g., particle size distributions at different reaction times) with static or final process data (e.g., optical properties of the final NP dispersion). Lastly, as the process data serve as the ground truth for model calibration, their reliability is critical. Outliers or experimental deviations are interpreted as genuine physical effects and will reduce the quality and thus the predictive character of the model. A highly reproducible synthesis route is thus imperative for accurate modeling.

#### 3.1.2 Structure Optimization

The property function (Figure 2B, left) connects the structure of nanoparticulate materials to their resulting macroscopic properties. While the term structure is often associated with large‐scale assemblies, such as colloidal crystals or other photonic architectures, it can also refer to the nanoscale dispersity of individual particles. For instance, the shape, size, and composition of single plasmonic NPs can be tailored to achieve specific optical effects, such as resonance positions or defined transmission colors. In the following, we highlight three instructive examples of structure optimization, focusing on the optimization of the composition of symmetric, multimaterial NPs, the shape of anisotropic particles, as well as the structure of assemblies of individual NPs. These different geometries highlight the wide playground of structural properties of nanomaterials that influence the resultant properties. Notably, as each of these systems focuses on a particular aspect (composition, shape, and assembly), they require different frameworks for structural optimization. The selection therefore aims to introduce general concepts of structure optimization that can be adopted to different materials, geometries, or assemblies, with properties that can be optimized to a desired application.

##### 3.1.2.1 Structure optimization of isotropic, multimaterial nanoparticles with plasmonic properties

To introduce how the structure of single particles can be optimized for a desired property, we exemplarily discuss the optimization of isotropic, multimaterial particles, taking the example of individual silica core–gold shell particles, which are optimized to display a predefined plasmonic color [152]. In this work, multilayer Mie theory simulations [177] were used to derive a model predicting the optimal core size and shell thickness of gold‐coated silica NPs to achieve a desired transmission color. The predicted structures were subsequently used as target structures and synthesized using a continuous‐flow microreactor. This example is fully knowledge‐based in that the desired property is described by Maxwell's equations in the form of Mie theory, which yields the extinction spectrum and therefore the color of the dispersion from first principles if the material properties (complex refractive index and sizes) of the particles are known.

In the first step, the accessible color space was identified, that is, the range of colors that can be achieved via a given material system. This color space was mapped in CIELAB coordinates and was constructed by simulating various combinations of silica core diameters, shell thicknesses, and particle concentrations. Figure 4A‐i illustrates the resulting 3D color space for silica‐gold core–shell NPs.

**FIGURE 4:**
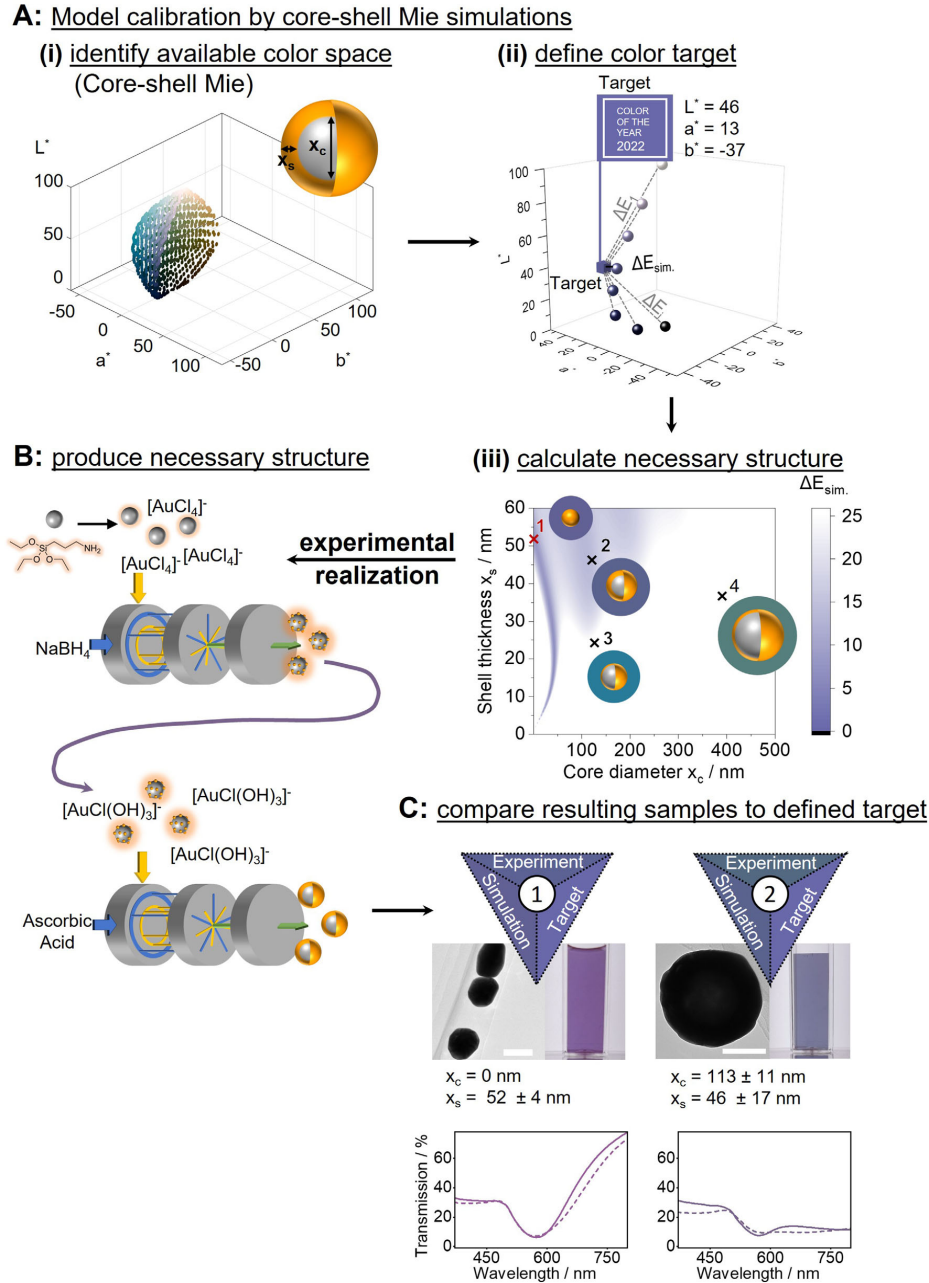
Guide for the inverse design of a targeted plasmonic color, using silica core–gold shell NPs optimized via a knowledge‐based property function, as outlined in Figure 2. (A) The theoretically available color space of the system (here: gold shells on silica core particles) is calculated via Mie simulations (i). A color target is defined (ii) and the corresponding NP structure (i.e., shell thickness *x*_s_ and core diameter *x*_c_) is identified (iii). (B) The predicted NP structure is experimentally realized using a micro‐mixer setup to produce particles with the desired disperse properties. (C) For validation, the experimentally obtained properties (here: transmission colors, transmission spectra, and final NP dispersity) are compared to the predicted values generated via the property function (insets). Reprinted from Journal of Colloid and Interface Science, Vol. 691, G. Bleyer, A. Uihlein, U. Sultan, M. Ickler, R. Yamashita, L. Pflug, S. Watanabe, and N. Vogel, “Predictive design of plasmonic color,” 137408, Copyright (2025), with permission from Elsevier [152].

Within this space, a desired target color was selected, defined by a combination of L*, a*, and b* values (Figure 4A‐ii). The model then computed the optimal particle structure to provide the desired color, by varying the core diameter, shell thickness, and NP concentration in dispersion. The color difference between the simulated color of a given structure and the color target in the CIELAB color space served as the objective function, defining the “closeness” of a color to the predefined target. A lower color difference indicates a better match between the simulated NP color and the target color. The symmetric nature of the chosen particle system enabled the simulation of the entire color space as a function of particle properties, thus providing color difference maps that determine global and local optima in particle structure for a desired color. Figure 4A‐iii exemplarily shows such a color map that describes the closeness of dispersion colors to the Pantone color of the year 2022. Selected particle structures at different locations on the color map and their corresponding transmission colors are illustrated. Particle structure 1 shows the global minimum, that is, the optimal particle structure to yield the desired color. Particle structure 2 exemplarily shows a structure within a local minimum, showing a slightly higher deviation in color, but located within a broad minimum that may be more robust toward experimental size distributions. Points 3 and 4 show “off target” regions where the particle structure does not yield colors that match the target.

With the optimal target structure defined (particle structure 1 in Figure 4A‐iii), the corresponding NPs were synthesized using a continuous‐flow microreactor (Figure 4B). After synthesis, the resulting experimental NP structures were compared to both the predicted structures and their transmission color compared to the color target. Figure 4C shows the agreement between the simulated and the experimentally observed colors (see insets (1) and (2), corresponding to the same points in the color difference map in Figure 4A‐iii). The close agreement of the transmission spectra in Figure 4C further supports the match of simulated (dashed lines) and experimentally realized (solid lines) optical properties. For the optimal structure (inset (1)), both the simulated and experimentally produced colors align well with the target color. The small deviations can be attributed to polydispersity effects, that is, distributions in core diameter and shell thickness that are not included in the model.

##### 3.1.2.2 Structure optimization of anisotropic nanoparticles with magnetic properties

Altering the shape of NPs vastly increases the degrees of freedom to design NPs, and thus provides a wide playground to tailor the resultant properties [178, 179, 180, 181, 182]. For instance, shape anisotropy can introduce additional plasmon modes and enable spectral tuning into the near‐infrared for photothermal applications [178], generate electromagnetic hot spots at highly curved tips for enhanced Raman sensing [179], or expose specific crystallographic facets that alter catalytic activity and selectivity [181]. An instructive example for the structure optimization of anisotropic NPs is the design of magnetite nanodiscs with a defined magnetization configuration [167]. Disc‐shaped magnetic particles can adopt three distinct equilibrium spin arrangements, that is, a vortex state, in which the spins close into a flux loop, an in‐plane state, and an out‐of‐plane state. Which of these is realized is dictated by the geometry of the particle, specifically by the combination of its diameter and thickness. The magnetization configuration therefore represents a macroscopic property of the particle system that is determined by its disperse properties, in direct analogy to the transmission color of the plasmonic systems discussed in Section 3.1.2.1. In this study, the vortex state was selected as the target property. Its appeal lies in the fact that particles in the vortex configuration carry a near‐zero net magnetic moment at zero field, which suppresses magnetic attraction between particles and thus provides excellent colloidal stability. Yet they acquire a large induced magnetic moment and switch to in‐plane magnetization once a weak external field is applied. Particles with a vortex ground state are consequently able to transduce weak, slowly varying magnetic fields into mechanical torque, which the authors subsequently exploited to remote control mechanosensory neurons. The design task is thus to produce NPs with a structure yielding a vortex state.

To do this, a knowledge‐based property function was constructed from micromagnetic simulations using the Object Oriented MicroMagnetic Framework (OOMMF) [183]. Hexagonal discs were discretized into volume elements, each assigned a random initial magnetization direction, and the equilibrium spin configuration was obtained by energy minimization. Since the outcome depends on the initial conditions, each geometry was simulated repeatedly, and the resulting configurations were averaged and classified into the three equilibrium spin arrangements discussed above, that is, in‐plane, out‐of‐plane, and vortex. Evaluating this procedure across a range of diameters and thicknesses yields the phase diagram shown in Figure 5A‐ii, which assigns a magnetization configuration (Figure 5A‐i) to every point in the structural design space. This map effectively forms the property function of the system, derived from knowledge‐based simulations. It differs from the color targets discussed in Section 3.1.2.1 in that the design problem is not the minimization of a distance metric but the identification of the region in which the desired state is realized.

**FIGURE 5:**
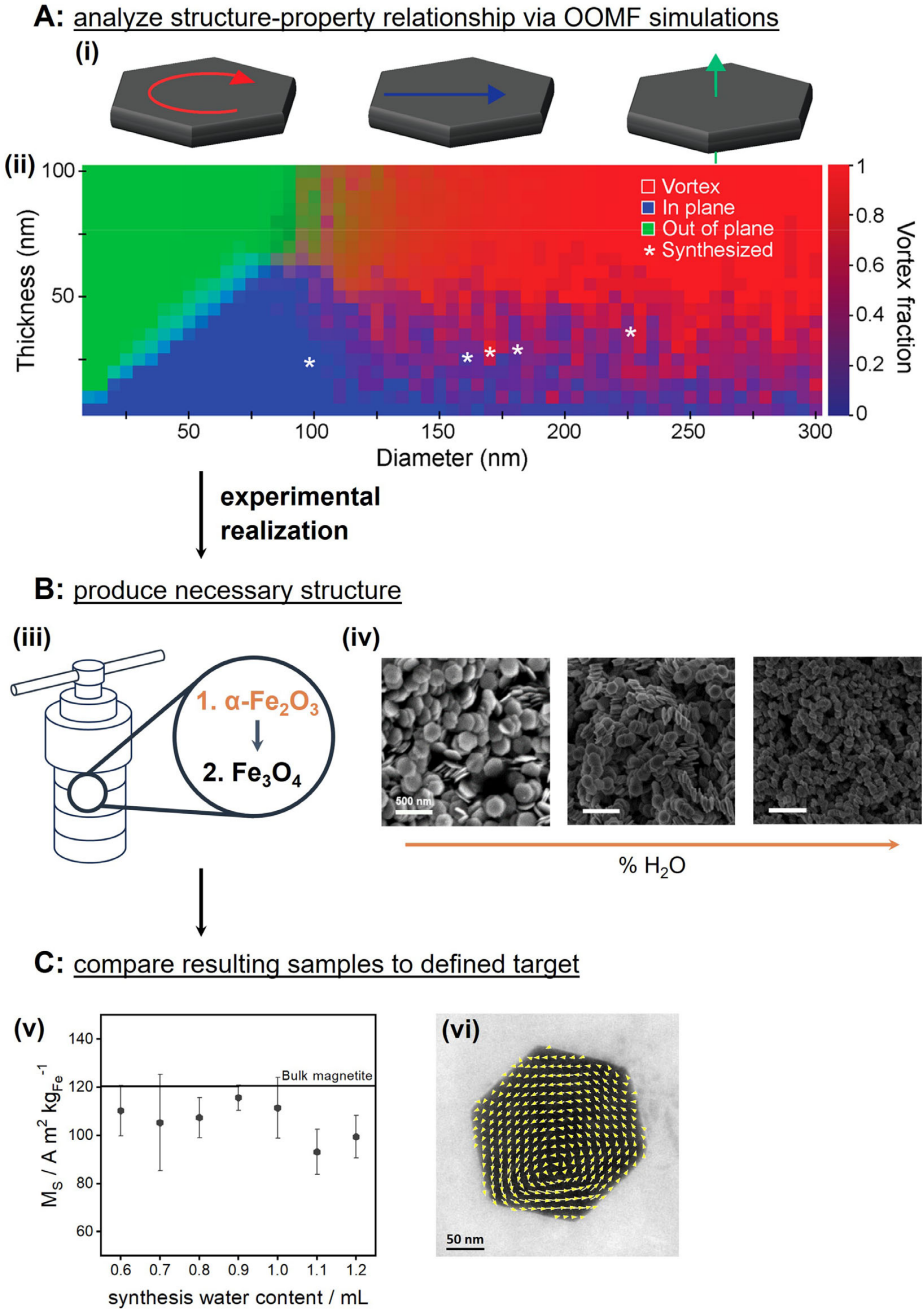
Guide for the inverse design of magnetic nanodiscs with a targeted magnetization configuration, via structure optimization using a knowledge‐based property function, as outlined in Figure 2. (A) The structure–property relationship of the system is calculated via micromagnetic (OOMMF) simulations, yielding a phase diagram that assigns the equilibrium magnetization configuration (vortex, in‐plane, or out‐of‐plane (i)) to each combination of disc diameter and thickness (ii). Used with permission of the American Chemical Society, from “Magnetic Vortex Nanodiscs Enable Remote Magnetomechanical Neural Stimulation”, D. Gregurec, A. W. Senko, A. Chuvilin, P. D. Reddy, A. Sankararaman, D. Rosenfeld, P. Chiang, F. Garcia, I. Tafel, G. Varnavides, E. Ciocan, and P. Anikeeva, Vol. 14(7), 2020 [167]; permission conveyed through Copyright Clearance Center, Inc. (B) Some predicted structures (see asterisks in (A‐ii)) are experimentally realized via a two‐step solvothermal route, in which hematite nanodiscs are crystallized and subsequently reduced to magnetite (iii). The disc diameter is controlled via the water content of the reaction mixture, as shown by the SEM images of the resulting particles (iv). (C) The experimentally obtained properties are compared to the defined target. Saturation magnetization of the synthesized discs as a function of water content used in the synthesis, referenced against the bulk magnetite value (v) and the magnetic vortex configuration of individual particles resolved by electron holography (vi).

With the target property defined as the vortex configuration, the phase diagram is read backwards to extract the disc geometries that support this configuration. Five combinations of diameter and thickness were selected for experimental realization (asterisks in Figure 5A‐ii), spanning diameters between roughly 100 and 250 nm. These structures were subsequently synthesized via a two‐step solvothermal route (Figure 5B‐iii). Hematite (α‐Fe_2_O_3_) nanodiscs were first synthesized from an iron chloride precursor in an ethanol‐water mixture and subsequently reduced to magnetite (Fe_3_O_4_) under a reductive hydrogen atmosphere. This reduction step largely preserves the disc geometry while converting the nonmagnetic hematite phase into the magnetic magnetite. The structural handle in the synthesis is the water content during hematite formation. Water molecules act as a binder between the terminating oxygen atoms of the hematite [0001] plane and ethanol or acetate, inhibiting growth along the corresponding crystallographic direction so that increasing the water fraction progressively reduces the disc diameter (Figure 5B‐iv).

The synthesized NPs are finally compared to the design target on multiple levels (Figure 5C). First, the saturation magnetization measured across the size series approaches the bulk value of magnetite (Figure 5C‐v), confirming both the near‐complete conversion of the hematite precursor and the fact that the discs magnetize as expected in an applied field, which in turn validates the material parameters entering the simulation. Second, the magnetic spins of individual particles were resolved by electron holography, which visualizes the magnetic configuration (Figure 5C‐vi), providing evidence that the targeted vortex state was realized. Lastly, a corresponding magnetization map confirms magnetic flux density confined to the interior of the disc. The particles are therefore magnetically neutral to the outside, as required for their colloidal stability.

This example instructively demonstrates the workflow established in Figure 2. A simulated structure–property relationship based on physico‐chemical relationships, maps particle structure onto the resulting magnetization configuration, the desired configuration is defined as the target, the corresponding structure is extracted from the model, and the synthesized particles are verified against the prediction.

##### 3.1.2.3 Structure optimization of assemblies of individual particles with photonic properties

Structure optimization can also be extended to assemblies of individual particles, which, provided they are sufficiently similar in size, can form ordered assemblies termed colloidal crystals [184]. If such colloidal crystals are made from primary particles with dimensions within the size range of visible light waves, they can form photonic structures that exhibit pronounced structural coloration [120, 122, 124, 125]. The inverse design of such colloidal photonic crystals for optimized color properties has been demonstrated using a knowledge‐based property function [151]. In this study, polystyrene core‐polydopamine shell (PS@PDA) particles were used as primary particles [148, 149, 150, 185], where the PDA shell thickness and PS core size could be independently adjusted to tune absorber content and reflection color, respectively. PDA, a synthetic analogue of the naturally occurring pigment melanin, acts as an optical absorber that reduces the overall reflectivity of the sample. In particular, structural defects within the crystal lattice cause diffuse light scattering, thereby lowering color saturation and imparting a whitish hue to the targeted color [143, 186]. To mitigate this detrimental effect, light‐absorbing materials can be incorporated into the photonic crystal structure. These absorbers reduce incoherent scattering from defects and irregularities [143, 145, 146, 185, 187, 188], effectively purifying the photonic stop band and enhancing color intensity. Several studies have shown that increasing the absorber content yields more saturated and well‐defined colors [124, 144, 187].

The inverse design to directly obtain optimal absorber contents was realized by the inversion of a knowledge‐based property function. Specifically, the chroma (i.e., the color intensity) of the resulting structural color was first optimized within a digital model system (Figure 6A) by allowing the optimizer to vary the absorbing properties within the particle structure. The predicted optimal configuration was then experimentally realized (Figure 6B), yielding an optimized material without the need for experimental parameter variations.

**FIGURE 6:**
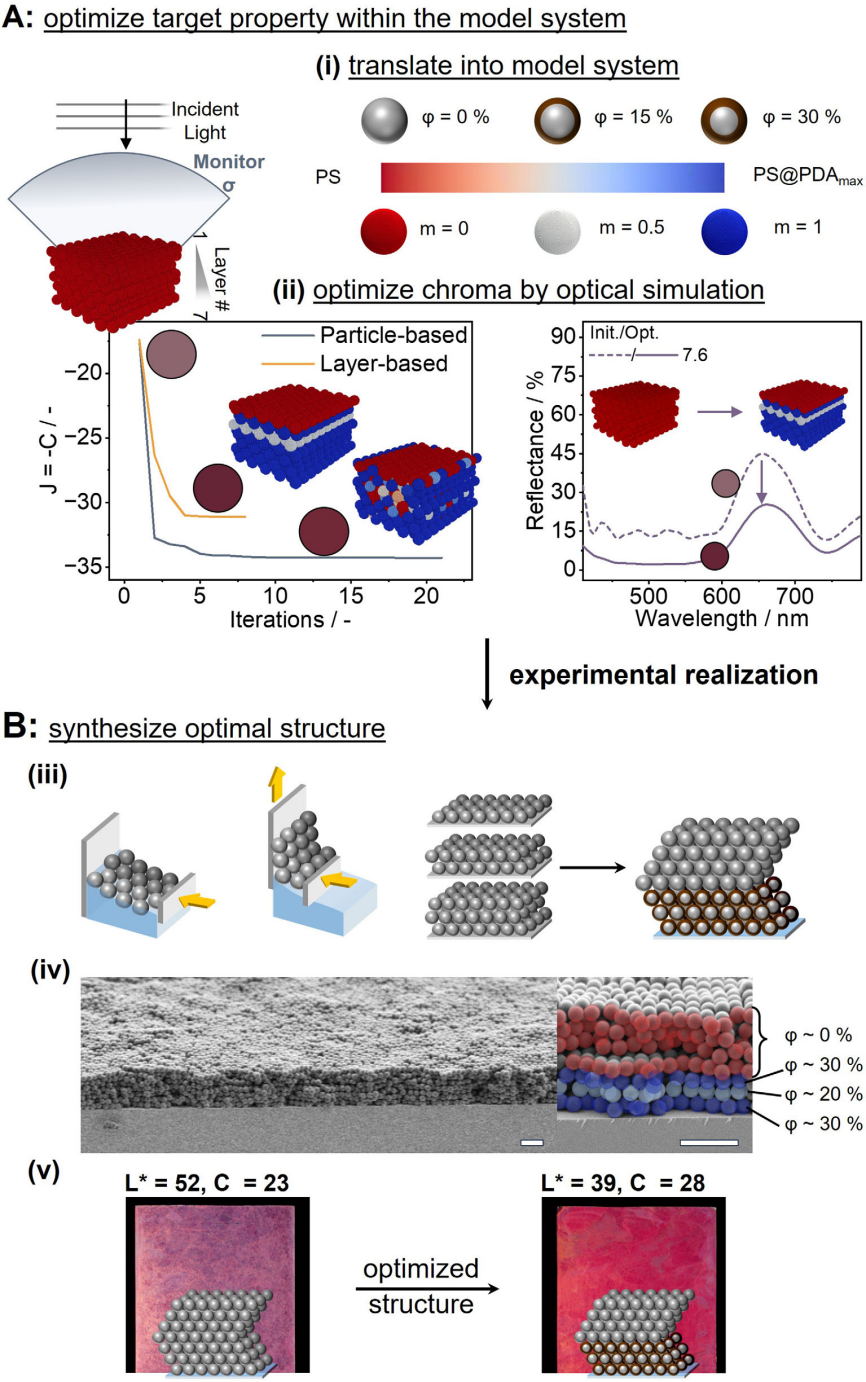
Guide for the inverse design of colloidal photonic crystals with optimized structural coloration, via structure optimization using a knowledge‐based property function, as outlined in Figure 2. (A) The experimental system, consisting of polystyrene@polydopamine core–shell particles is translated into a digital model (i), in which the optical properties of the particles with varying absorber volume fractions are incorporated. A target property (here: chroma of the resultant macroscopic structural color) is optimized by systematically varying the absorber content (*φ*) and spatial distribution within the crystal lattice (ii‐left). An optimal structure is found based on simulated reflectance spectra (ii‐right). (B) The predicted structure is experimentally realized using a layer‐by‐layer assembly method in a Langmuir trough (iii), structurally characterized in the SEM (iv), and compared to the unoptimized structure based on its structural color (v). Reproduced from Ref. [151], DOI: 10.1002/adom.202403279, under the terms of the CC BY 4.0 license (https://creativecommons.org/licenses/by/4.0/). Panels were cropped and rearranged.

In the model, the PS@PDA primary particles were represented by spherical particles with tunable sizes and effective refractive indices combining polystyrene and polydopamine in their respective volume fractions to reduce computational costs. Figure 6A shows the digital twin of a colloidal photonic crystal within the simulation setup. In this representation, blue particles represent the highest absorber content, whereas red particles correspond to pure PS without absorber (Figure 6A‐i). The optical properties of the colloidal photonic crystal were simulated using the discrete dipole approximation method (DDA) [189] to obtain the optical reflection spectra and, after transfer to the CIELAB color space, the resulting structural coloration (Figure 6A‐ii).

A mathematical structure optimization algorithm subsequently varies the absorber content φ of each individual particle or each individual layer of particles (Figure 6A‐ii, left). To identify an optimal structure, an objective function is defined, which, in its simplest form, maximizes the resulting chroma. Additional terms can be incorporated into the objective function to account for tradeoffs between high chroma values and color lightness, that is, to optimize for a desired lighter or darker shade of a hue [151]. The optimizer converges on an ideal structure with highest chroma (i.e., lowest *J* values). As shown in Figure 6A‐ii, a particle‐resolved structure optimization, where the optimizer is allowed to change the refractive index of each individual particle, yields a higher chroma, while the layer‐based optimization converges quicker but to lower chroma values. This tradeoff is justified as experimentally, it is not possible to specify the position of individual particles, but layers of different composition can be stacked to form an experimental realization of the layer‐based optimization [186].

The resulting optimized structure exhibits significantly increased chroma due to reduced reflectance at off‐peak wavelengths (Figure 6A‐ii, right). Interestingly, the optimizer achieves the highest chroma by preferentially placing absorbing particles in the lower layers (inset, Figure 6A‐ii, right). This not only enables the design of structures with improved optical properties but also allows comparisons with structural arrangements in nature. For example, the *Morpho* butterfly's color is enhanced due to the placement of light absorbing pigments below the nanoscale lamella structures on its wings [151].

The blueprints obtained from the structure optimization were subsequently experimentally realized via layer‐by‐layer assembly using a Langmuir trough (Figure 6B‐iii). Colloidal monolayers formed by particles with the identified optimal absorber content are assembled at the air–water interface [190, 191], compressed into close‐packed arrangements, and transferred onto a substrate [151, 186]. This process is repeated to build a seven‐layer structure, equivalent to the simulated design (Figure 6B‐iv), allowing precise control over the spatial distribution of absorbers. Finally, a comparison of the optimized photonic crystal with an absorber‐free structure clearly shows a substantial increase in color saturation (Figure 6B‐v). It should be noted that the model optimizes a single ideal structure and does not capture structural imperfections or lattice defects that inevitably arise during self‐assembly, which represent an additional source of variability in the optical response of the experimentally realized crystal.

#### 3.1.3 Integrated Property‐Process Optimization

Even when considered separately, property and process functions already enable the inverse design of nanoparticulate materials. However, full inverse optimization approaches are even more powerful, as they allow the identification of synthesis conditions that yield particle systems with optimized macroscopic properties. These approaches require a full property–process relationship, that is, a model that directly links the relevant process parameters to the resulting product properties. This was recently addressed in a study on bimetallic AgAu alloy NPs [39]. Based on a developed mechanistic understanding, a knowledge‐based model connecting key process parameters to the optical properties of the resultant particles was derived and calibrated. This model was used not only to validate the proposed formation mechanism but also to gain additional process insights and to identify optimal process conditions for achieving a desired optical property of the macroscopic particle dispersion.

As a first step, a mechanistic understanding of the alloy NP formation was established from detailed studies of the formation process. In short, the process comprises the precipitation of silver chloride (AgCl) crystals, reduction of silver and gold precursors, homogeneous and heterogeneous nucleation of the final alloy NPs, the formation of core–shell intermediate structures, and their structural reorganization into homogeneously alloyed NPs. The steps are evident from STEM images (Figure 7A‐iii), the evolution of extinction spectra (Figure 7A‐i), and the mean particle diameter as a function of the molar gold content (Figure 7A‐ii). Based on these complementary observations, the complete formation mechanism can be reconstructed (see Figure 7B).

**FIGURE 7:**
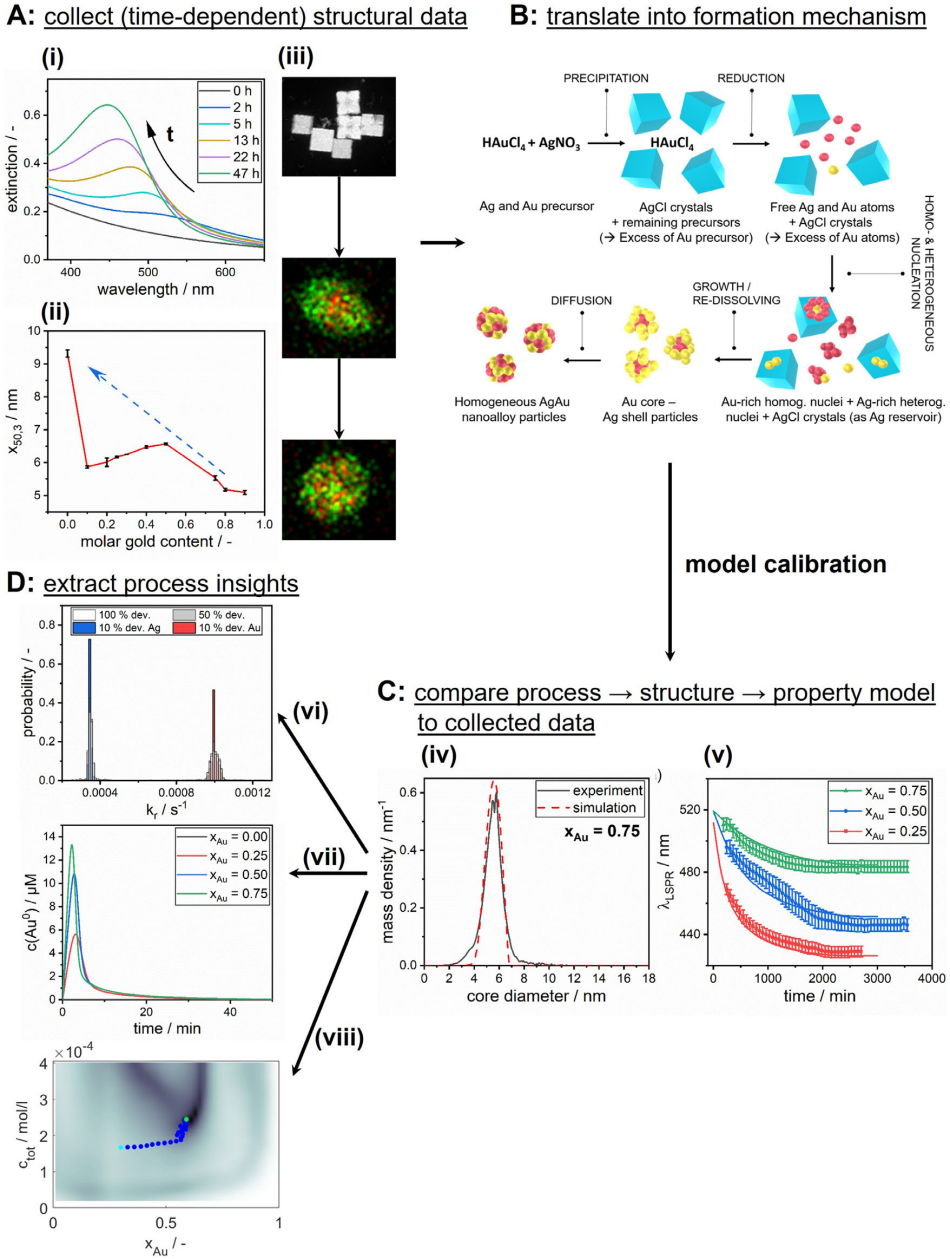
Guide for the inverse design of silver–gold alloy nanoparticles via a knowledge‐based full property–process relationship, as outlined in Figure 2. (A) Time‐resolved structural data of silver–gold alloy nanoparticles are collected using both in situ and ex situ methods, including in situ extinction spectroscopy (i), ex situ analytical ultracentrifugation (AUC) measurements (ii), and ex situ STEM‐EDX measurements (iii). (B) Based on the collected data, a detailed formation mechanism is derived and modeled using physically grounded equations. The model is calibrated using the collected experimental data to establish a full property–process relationship. (C) The model's predictions are validated against experimental results, including full particle size distributions at the end of the reaction for different final molar gold contents (iv) and resonance peak positions over time (v). (D) Process insights such as experimentally inaccessible process parameters (here: reaction rate constants (vi) and gold atom concentration profile (vii)) can be extracted from the calibrated model. Additionally, the property–process relationship can be used to extract optimal process conditions to achieve a desired NP structure based on defined optical response colors (viii). In this example the concentrations of silver and gold precursors are treated as adjustable process parameters. For a defined optical target, the model computes the optimal concentrations. Reproduced from Ref. [39], DOI: 10.1016/j.cej.2024.149429, under the terms of the CC BY 4.0 license (https://creativecommons.org/licenses/by/4.0/). Panels were cropped and rearranged.

With knowledge of the formation mechanism, a two‐dimensional (2D) PBE model of the key steps in the formation process was developed [39]. The model captures the structural evolution of AgAu alloy NPs over time, that is, the dynamic evolution of the 2D distribution in both particle size and composition. To translate this evolving structure into corresponding macroscopic properties, Mie theory was applied to compute the optical properties as a function of particle size and composition. The ensemble‐averaging effect is thereby directly incorporated into the model, as the optical response is computed from the full 2D distribution in particle size and composition rather than from a single representative mean structure. This step bridges the gap from process parameters to the macroscopic properties of the formed particles, thereby completing a full description of the property–process relationship. The model was then calibrated using experimental data, and the simulated results were compared to experimental measurements (see Figure 7C). The quantitative agreement for both the structural properties of the formed particles, for example, PSDs at different molar gold contents (Figure 7C‐iv), and the macroscopic optical properties, in particular, the evolution of the LSPR wavelength over time for different final gold contents (Figure 7C‐v) demonstrates that the model accurately captures the particle formation process [192].

This model can now serve to extract experimentally inaccessible process parameters (in analogy to the process model in Section 3.1.1) and thus enable a deeper understanding of the reaction process itself. These parameters include growth rate constants, Mersmann constants (Figure 7D‐vi), and atomic concentration profiles over time (Figure 7D‐vii), which are nearly impossible to measure experimentally. Conversely, the model also provides the foundation for the inverse design of bimetallic AgAu alloy NPs. Within this framework, a desired transmission color or plasmon resonance position can be freely defined as the target property. Based on this specification, the knowledge‐based model determines the process conditions necessary to synthesize AgAu alloy NPs with the desired optical properties via a directly coupled property–process function. An exemplary result is shown in Figure 7D‐viii). In this example, the total metal precursor concentration *c*_tot_ and the molar gold fraction *x*_Au_ are treated as process conditions to be optimized. For a defined transmission color with L* = 70, a* = 30 and b* = 100, the model determines optimal values for the total metal concentration and the molar gold fraction in the synthesis solution that yield the specified optical response. A derivative‐based optimization algorithm is employed, where the cyan dot in Figure 7D‐viii marks the starting point and the green point indicates the optimal solution identified by the optimizer. The background shading represents the color difference between the colors obtained under the respective process conditions and the target color, with darker shades corresponding to smaller deviations. The resulting landscape exhibits a highly irregular structure with an elongated minimum between molar gold fractions of 0.6–0.7 and total metal concentrations of 2–4∙10^−4^ molL^−1^, wherein the global minimum is located. Such advanced process control is achievable only through a mathematically defined property–process relationship to guide the process.

Building a full knowledge‐based property–process relationship that links synthesis parameters via the nanoparticle structure to a macroscopic property in a single optimizable model remains an open challenge for nanoparticulate systems beyond the optical properties presented above. While separate process and property functions for a variety of target particles exist, their combination into an end‐to‐end predictive model has not yet been achieved. A recent contribution suggests this goal is within reach: Uhlemann, Peukert et al. [193] outline how population balance models, dissolution kinetics, and physiological absorption models can in principle be combined to predict in vivo pharmacokinetics directly from synthesis conditions for nanoparticulate drug formulations. This further shows that the framework established in this perspective is more broadly applicable to functional nanoparticulate systems in other fields of application.

### 3.2 Data‐Based Optimization

Data‐based optimization does not require prior knowledge of the underlying physics of a system. Instead, the parameter space is systematically explored through datasets provided by prior experiments. An algorithm then establishes a heuristic mapping between input and output parameters, allowing the prediction of suitable process or structure parameters. Because these relationships are often nonlinear [38], many optimization problems necessitate multivariate optimization, and experimental design is commonly guided by design of experiments (DoE) techniques [76, 194]. To establish the link between input and output variables, a range of mathematical approaches has been explored in the literature. These include traditional regression techniques such as polynomial regression [76], as well as more advanced machine learning (ML) methods, including random forests and support vector machines [195, 196]. In recent years, deep learning models based on artificial neural networks (ANNs) have also gained increasing attention [197].

Such data‐based optimization has been applied for different nanoparticulate systems [38, 76, 195, 196, 197, 198, 199, 200, 201, 202, 203, 204, 205, 206, 207, 208, 209, 210, 211, 212, 213, 214, 215, 216, 217]. For process optimization existing approaches include tuning the synthesis conditions for silver nanoplatelets [196] and AgAu alloy NPs [76] to achieve specific transmission colors, as well as employing ANNs to control the structure of titanium dioxide (TiO_2_) nanocrystals [38], employing a data‐based process function to simultaneously optimize crystal phase, particle size, and polydispersity of colloidal cobalt oxide (CoO) NPs for CO_2_ hydrogenation catalysis [211], and integrating active learning with robotic automation to screen a large formulation design space to predict particle size, polydispersity and drug loading as process outputs [210]. Structure optimization has been applied to the inverse design of structural color based on dielectric silicon dioxide (SiO_2_) ring and pyramid arrays [201], melanin@SiO_2_ particle assemblies [202], or the selection of materials in multilayer thin films [203]. In addition, such methods were employed in the development of high‐performance structural color filters [204, 205, 206], the optimization of plasmonic coloration of metal surfaces via laser‐induced ablation [200], or to the modeling of catalytic performance in platinum or RhAu alloy NPs [195, 213, 214]. Full property–process relationships have also been applied to nanoparticulate systems targeting different functional properties, including optical, catalytic, and antibacterial applications [76, 216, 217]. For instance, Furxhi et al. demonstrated training regression models on literature‐extracted data to directly connect silver nanoparticle synthesis parameters to their antibacterial efficiency [216]. It is worth noting that such integrated data‐based property–process relationship models are comparatively rare relative to the more abundant isolated process and property functions, likely reflecting the prevailing data scarcity in the field.

Data‐based inverse design has been an active research direction within the broader photonics and nanophotonics community as well [218, 219, 220, 221, 222, 223, 224]. This includes applications ranging from topology optimization of photonic devices [221] to artificial neural network‐based inverse design of multilayer and core–shell NPs [220, 222, 223] and plasmonic structures [218], as well as more recent physics‐informed approaches targeting specific spectral responses [219, 224].

In general, in the context of inverse design of nanoparticulate materials, the nature of the input parameters depends on the application of the data‐based model. Input parameters may correspond to process conditions, such as temperature, pressure, or precursor concentrations, or to structural features such as characteristic lengths, refractive indices, or surface charges. Thus, in analogy to the knowledge‐based approaches, data‐based models can serve as process functions when process parameters are linked to particle structure, as property functions when structural parameters are related to macroscopic properties such as color or catalytic activity, or as full property–process relationships when process parameters are directly connected to the resultant macroscopic product performance of the formed nanoparticulate systems.

#### 3.2.1 Process Optimization

An instructive example for data‐based process optimization is the inverse design of titanium dioxide (TiO_2_) nanocrystals synthesized via a hydrothermal process by employing an ANN model [38]. The ANN relates process parameters to the resulting particle size, polydispersity, and shape, and the iterative retraining of the ANN with new experimental data can enhance the model's accuracy over time.

In the first step, the parameter space is defined based on existing process literature. The selected input parameters are the process temperature (*T*), the initial pH, and the concentrations of the triethanolamine titanium complex Ti(TeoaH)_2_, used as the titanium precursor, and the shape‐controlling agent triethanolamine (TeoaH_3_). The output variables are the hydrodynamic radius of the produced nanocrystals, their polydispersity, expressed as a percentage of the hydrodynamic radius, and their aspect ratio, determined from fitted ellipsoids. By including polydispersity as an output parameter, the model directly captures the width of the size distribution as an explicit design objective alongside the mean particle dimensions, an important consideration for the quality of the resultant particle system. To generate training data, experiments are performed across the defined input parameter space. To efficiently sample the space, design of experiment methods in the form of the Box–Wilson central composite design are employed (Figure 8A). The resultant nanocrystals are analyzed in terms of their structure and three separate ANN models, one for each output variable, are then trained using these experimental data. Each ANN shares the same architecture, comprising four input neurons (one per process parameter), one hidden layer with three neurons, and one output neuron for the respective output variable (Figure 8A, inset). The network consists of 15 interconnections, uses a sigmoidal activation function, and is trained via backpropagation, resulting in an initial model. This initial ANN model is validated against an independent set of experiments, which demonstrated acceptable predictive performance and confirmed the suitability of the ANN approach to model the process function (Figure 8B). The authors then adopt an iterative strategy where they supplement the training set with the validation data and retrain the ANN, yielding a refined version of the model. This iterative retraining is repeated multiple times throughout the study, gradually increasing the number of training data and improving the accuracy of the prediction (Figure 8C).

**FIGURE 8:**
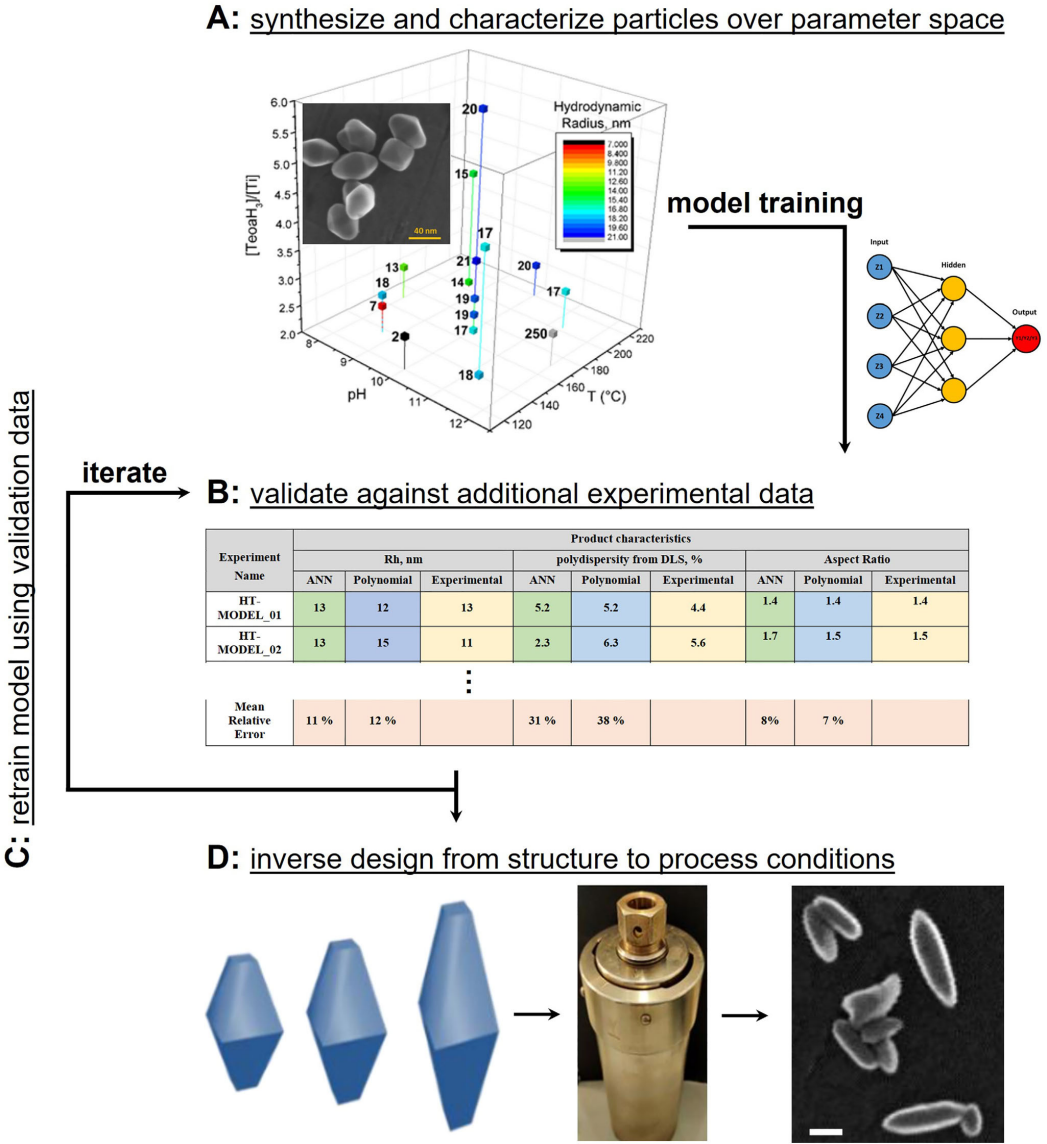
Guide for the inverse design of TiO_2_ nanocrystals via a data‐based process function, as outlined in Figure 2. (A) Hydrodynamic radii of TiO_2_ nanocrystals synthesized under various combinations of process parameters (*T*, pH, c(Ti(TeoaH)_2_), c(TeoaH_3_)) plotted over the respective input parameter values, with an exemplary SEM image shown in the inset. These data serve as the training dataset for multiple artificial neural networks (ANNs) correlating the aforementioned input parameters to one specific output parameter. (B) Comparison of experimental values for hydrodynamic radii, polydispersities, and aspect ratios with predictions from the trained ANNs and a polynomial regression model validating the predictive performance of the ANN model. (C) Experimental validation data are added to the training dataset in an iterative retraining process, continuously refining and improving the initial model. (D) The final ANN is used for the inverse design of TiO_2_ nanocrystals, predicting ideal process conditions that yield a targeted nanocrystal morphology. Specifically, nanocrystal target morphologies are defined (left) and synthesized in a hydrothermal reactor (middle) according to the predicted process conditions. Reproduced from Ref. [38], DOI: 10.1038/s41598‐020‐75967‐w, under the terms of the CC BY 4.0 license (https://creativecommons.org/licenses/by/4.0/). Panels were cropped and rearranged.

With the trained models at hand, the authors proceed to extract process insights. The authors start with conducting a sensitivity analysis, identifying the initial pH and process temperature as the most influential parameters affecting the final particle morphology. They also uncover trends such as a correlation between lower pH or temperature and smaller hydrodynamic diameters. In contrast to knowledge‐based models, which can predict data beyond the experimentally measured parameters, data‐based models extract relationships directly from the available process data. Thus, although they do not generate additional or previously unknown information, such models are valuable to identify correlations within the measured data and thereby can substantially enhance the understanding of the underlying process.

Most importantly, the calibrated ANN models are finally used for an inverse design of TiO_2_ nanocrystals. Specifically, the authors define a target morphology comprising a minimal hydrodynamic radius (*Y*_1_), a polydispersity below 5% (*Y*_2_), and an aspect ratio of 1.5 (*Y*_3_), which is assigned to low‐truncated anatase nanocrystals (Figure 8D).

Since the three output variables are predicted by three separate models, the authors formulate a multi‐objective optimization problem to simultaneously optimize all three variables via objective functions comprising desired ranges or values for output parameters *Y*_1_ to *Y*_3_. Based on these optimization formulations, the authors applied a genetic algorithm (GA) for global optimization and successfully identified the process conditions required to produce TiO_2_ nanocrystals with the desired structural characteristics. As a result, the model predicts optimal values for process temperature, initial pH, and the concentrations of Ti(TeoaH)_2_ and TeoaH_3_ leading to the targeted specifications of the TiO_2_ nanocrystals. Particle syntheses performed at the respective optimal conditions then yield nanocrystals with properties matching the targeted structural characteristics.

#### 3.2.2 Structure Optimization

Data‐based methods can also be employed to predict optimized nanoparticle geometries for a desired macroscopic property. An instructive example of such data‐based structure optimization is the use of a machine learning framework to predict the structural parameters of rotated nanohole arrays (RHAs) with a desired circular dichroism.

Gold nanohole arrays are fabricated by colloidal lithography [225, 226], also known as nanosphere lithography [227], or shadow sphere lithography [228]. In this technique, a colloidal monolayer serves as a mask onto which thin layers of the desired materials are deposited. Based on the deposition angle and the shadowing effect of the monolayer template the deposition of nanostructure arrays on the underlying substrate is controlled [227, 228]. If the colloidal mask is not closely packed, nanohole arrays, consisting of small holes in a continuous metal film, are formed [226, 229]. In the example, the authors focus on RHAs, comprising two layers of gold and/or silver that are deposited under a defined rotation angle to give rise to a chiral character of the formed structures (Figure 9A). The geometric properties of these nanostructures are determined by multiple parameters, including the diameter of the templating spheres (*d*), their center‐to‐center distance (*P*), the incident angle of the deposited metal film (θ) and the azimuthal angle between the two depositions (φ), the metal layer thicknesses (*t*), as well as the choice of metal and the number of layers (see Figure 9A). The macroscopic optical properties of the formed nanostructure arrays are thus governed by the complex interplay between all these parameters. Attempting to optimize this multidimensional system using traditional trial‐and‐error methods would require a large number of samples and thus high experimental costs and effort. To address this challenge, the authors developed a structure optimization workflow that integrates geometric modeling, numerical simulation, and ML models.

**FIGURE 9:**
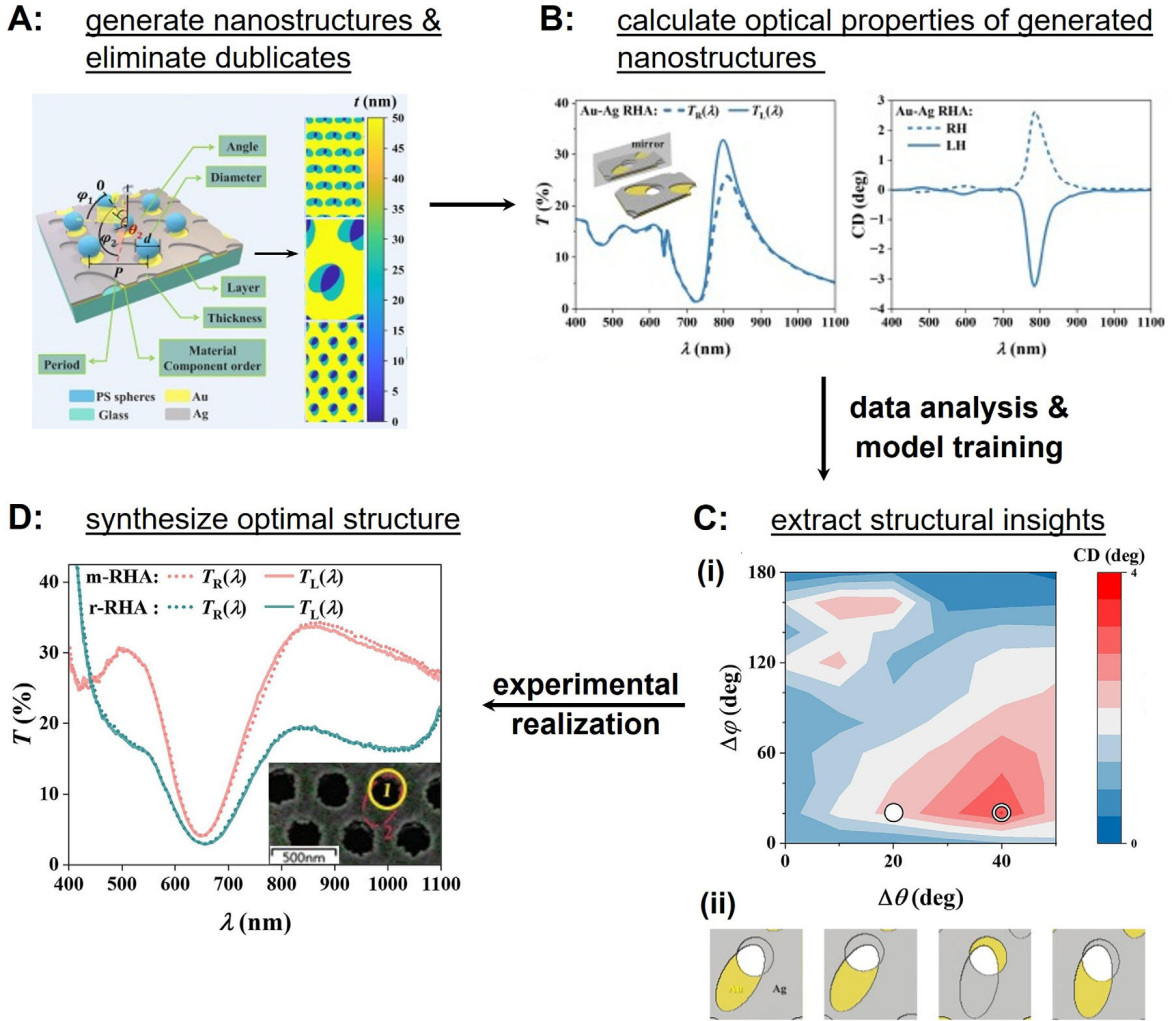
Guide for the inverse design of photonic silver–gold rotated hole arrays (RHAs) via a data‐based property function, as outlined in Figure 2. (A) Artificial RHAs are generated using a geometric model. (B) Calculated optical properties of the generated RHAs. The optical properties, together with the parameters of the geometric model are used as training data for a data‐based model. (C) The trained model enables structural insights, such as identifying values for design parameters which yield the best optical performance (i), as well as determining the optimal rotated nanohole array structure for a defined application (ii). (D) The predicted optimal structure is experimentally realized using shadow sphere lithography, characterized via STEM(‐EDX) analysis (inset), and evaluated for its optical response. Reprinted from Journal of Colloid and Interface Science, Vol. 680, Part B, X. Chen, M. Cheng, J. Zhang, Y. Wang, C. Chen, Q. Zhang, Y. Zhang, X. Wang, G. Zhang, and B. Ai, “A general optimization framework for nanofabrication using shadow sphere Lithography: A case study on chiral nanohole arrays”, 202–213, Copyright (2025), with permission from Elsevier [207].

In the first step, 3600 artificial RHA structures are numerically generated based on all possible combinations of the aforementioned structural parameters via a geometric model (Figure 9A). In a second step, a mathematical model eliminates redundant, structurally similar structures. This symmetry reduction decreased the number of unique configurations by 86%, significantly reducing the computational workload. Next, finite‐difference time‐domain (FDTD) simulations are used on the remaining structures to compute their transmission spectra under circularly polarized light. These were then converted into circular dichroism spectra, from which the peak circular dichroism intensities are extracted (Figure 9B). This allowed for a straightforward identification of optimal structural parameters corresponding to target optical properties (Figure 9C‐ii).

Using the simulated dataset, the authors subsequently analyzed the relationships between the design parameters and the resulting optical response. In particular, they identified clear correlations between the differences in incident and azimuthal angles of the two metal layers and maximum circular dichroism values (Figure 9C‐i). To further understand how each design parameter specifically influences the final optical properties, and thereby enable an inverse design strategy, the authors trained ML models to correlate all relevant structural parameters with the resultant circular dichroism intensity. They compared Categorical Boosting (CatBoost), Random Forest (RF), and Light Gradient Boosting Machine (LightGBM) models, with CatBoost showing the highest predictive performance. Consequently, CatBoost was employed in the third step of the workflow to assess the impact of individual fabrication parameters, where feature importance quantified how strongly a single parameter influences the optical output and permutation importance assessed the variation stability of each parameter on the resulting properties.

Finally, selected optimal structures were experimentally realized from the determined structural parameters (Figure 9D). While quantitative discrepancies between simulated and experimental circular dichroism values were observed and traced to experimental inaccuracies in structure geometries which are not captured by the model, the relative differences and performance trends among the RHAs were clearly reproduced. This experimental validation confirms the validity of both the geometric model (Figure 9A) and the data‐based structure–property relationship (Figure 9C).

#### 3.2.3 Integrated Property‐Process Optimization

Beyond modeling isolated process and property functions, data‐based models can also be employed to establish full property–process relationships, directly linking synthesis parameters to the macroscopic properties of the resulting nanoparticulate materials. In the following, we highlight two instructive examples of data‐based property–process relationships: One focused on the optical properties of silver–gold alloy NPs and one focused on the catalytic performance of silver‐doped manganese oxide particles.

##### 3.2.3.1 Integrated property‐process optimization of optical properties

Figure 10 shows the prediction of process parameters of AgAu alloy NP synthesis to yield a desired color target [76]. To create a basis for this inverse design, first Mie theory simulations, that is, a knowledge‐based property function, were used to compute the extinction spectra of spherical AgAu alloy NPs as a function of their structural properties, in particular particle size, composition, and concentration. These extinction spectra were then mathematically converted into transmission colors of the corresponding colloidal dispersions based on the CIELAB color space, which represents how the human eye perceives color (see Section 2).

**FIGURE 10:**
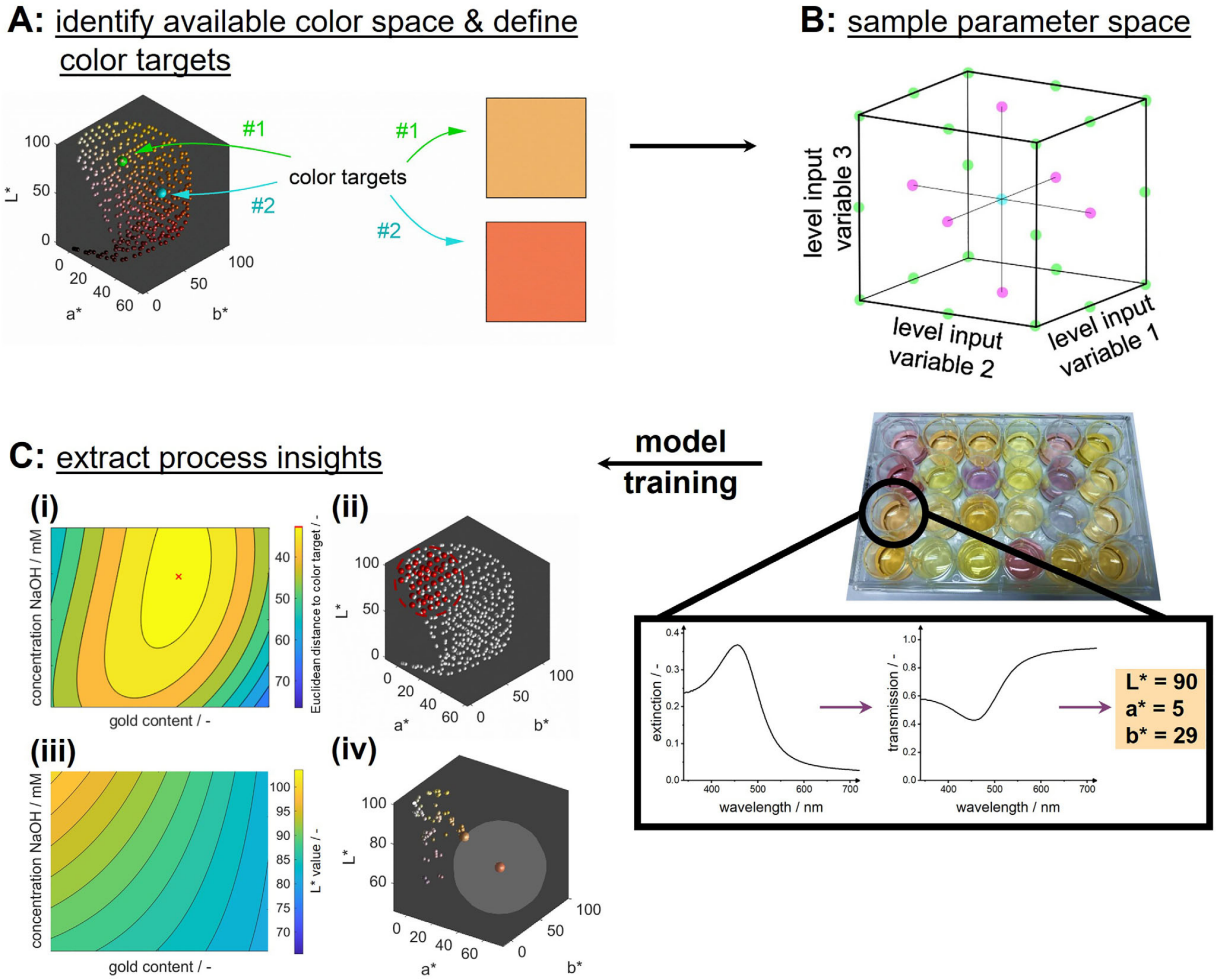
Guide for the inverse design of silver–gold alloy NPs via a data‐based full property–process relationship according to Figure 2. (A) The theoretically available color space for silver–gold alloy nanoparticles is identified via a property function and color targets defined within the available space. (B) The parameter space for relevant process parameters is systematically explored and sampled using design of experiment approaches and the macroscopic NP properties (here: transmission color) are collected. This combinatorial data set of process parameters (input) and transmission colors (output) is used to train a surrogate model (here: quadratic regression model). (C) Process insights such as process maps detailing which combination of process parameters yields the solution closest to the defined targets (i), the practically achievable portion of the theoretically available color space (ii), the evolution of the sample's L* value as a function of the process parameters (iii), and the closest, practically achievable color for a color target outside the practically achievable color space (color target #2) (iv) can be extracted from the trained model. Reproduced from Ref [76], DOI: 10.1039/d3na00856h, under the terms of the CC BY 3.0 license (https://creativecommons.org/licenses/by/3.0/).

The collection of these simulated colors defines the theoretically available color space for spherical AgAu alloy NPs. To perform inverse design, two color targets were selected with the goal to determine the process parameters required to match each target. Color target 1 was selected to be similar to colors resulting from the chosen synthesis route, that is, within the practically achievable portion of the theoretical color space, while target 2 was outside the practically accessible color space, shifted toward more saturated colors (see Figure 10A). Next, the process parameter space was sampled to generate training data for the data‐based model. As a synthesis protocol, a dextran‐based aqueous co‐reduction method was selected [110]. As relevant reaction parameters, the total metal precursor concentration (AgNO_3_ + HAuCl_4_), the molar ratio of gold precursor to total metal precursor (gold precursor ratio), the concentration of the pH‐adjusting agent (NaOH), and the concentration of dextran were selected. This four‐dimensional design space was explored using a structured design of experiment approach, including full‐factorial (FF), Box–Behnken (BB), and face‐centered central composite designs (CCD), all of which by design can resolve quadratic interactions between variables. Figure 10B exemplarily shows a visual representation of a full factorial design for 3 input variables. For each experimental condition, the extinction spectrum was determined and converted into the corresponding CIELAB color coordinates (Figure 10B). Because each extinction spectrum is measured from a colloidal dispersion, it inherently reflects the averaging over the full distribution of particle sizes and compositions present in the sample. The influence of structural dispersity is thus implicitly embedded in the training data and propagated into the model's predictions, without being explicitly parameterized.

Using these experimental data as input–output data sets, wherein the process parameters were chosen as the input parameters and the CIELAB coordinates as output, the authors developed and trained a regression model. Specifically, separate second‐order polynomial functions were defined for L*, a*, and b*. Since the trained model allows for the calculation of L*, a*, and b* values for any given set of process parameters, it enables the prediction of the resulting transmission color for each process condition. To evaluate how closely a predicted color matches the desired target, the Euclidean distance between the predicted and target color coordinates in the CIELAB space was calculated, which is often referred to as the color difference (see Section 3.1.2.1). This metric allows for the assessment of the suitability of process conditions for achieving a specified color target.

The trained regression model provides a range of process insights. First, and most importantly, it enables the inverse design of spherical AgAu alloy NPs by defining a color target and identifying the set of process parameters that minimizes the Euclidean distance between the predicted transmission color and the desired color target (Figure 10C‐i). Second, the model also allows assessment of whether a given synthesis route is suitable for producing NPs with the desired macroscopic properties. Although the theoretically accessible color space was defined at the beginning of the study, it is important to note that not all regions of this space are practically achievable with every synthesis method. In particular, some synthesis routes may be inherently limited in the range of achievable particle sizes or concentrations. By scanning the full theoretical color space using the trained model, it is possible to identify the subset that is practically achievable with the specific synthesis route used for model training (Figure 10C‐ii). Combined with the inverse design approach, the trained model therefore, after specification of a target color, enables assessment of whether that color is practically achievable and, if it is (color target 1 in Figure 10A), prediction of the required process conditions. If the color is not achievable for the chosen synthesis route (color target 2 in Figure 10A), the model predicts process conditions that yield transmission colors as close as the route can produce to the desired target (Figure 10C‐iv). Lastly, as the regression model comprises separate functions for the prediction of L*, a*, and b* values, it yields correlations between these values and the chosen synthesis conditions (Figure 10C‐iii). Thus, it also enables the optimization of a specific synthesis route for a defined color hue or lightness.

##### 3.2.3.2 Integrated property‐process optimization of catalytic properties

An instructive example showcasing how catalytic applications can be addressed in a data‐based optimization are silver‐doped manganese oxide (Ag/MnO_x_) catalysts for the oxidation of toluene to carbon dioxide (CO_2_) [217]. Here, the target property is the catalytic activity, quantified by T_50_(CO_2_), the temperature at which 50% of the theoretical CO_2_ yield is reached. A lower T_50_(CO_2_) corresponds to a more active catalyst and thus represents the objective function to be minimized. The activity itself emerges from the combined effect of specific surface area, the average oxidation state of manganese, and the density of surface oxygen vacancies, each of which depends on the synthesis conditions in a way that is not easily accessible to a physical description. A data‐based property–process relationship, connecting process parameters directly to the macroscopic property of the final product, is therefore the natural choice.

Four process parameters were identified as relevant for the catalyst synthesis: silver loading (*L*_Ag_), stirring time (*t*_s_), calcination temperature (*T*_c_), and calcination time (*t*_c_) (Figure 11A). This space was sampled using a central composite design, reducing the 625 combinations of a full factorial design to 30 representative experiments, including six repetitions of the center point to quantify the experimental error. Each of the 30 catalysts was synthesized by precipitation and its activity determined in a fixed‐bed reactor under a continuous toluene‐in‐air feed, yielding one T_50_(CO_2_) value per condition (Figure 11A).

**FIGURE 11:**
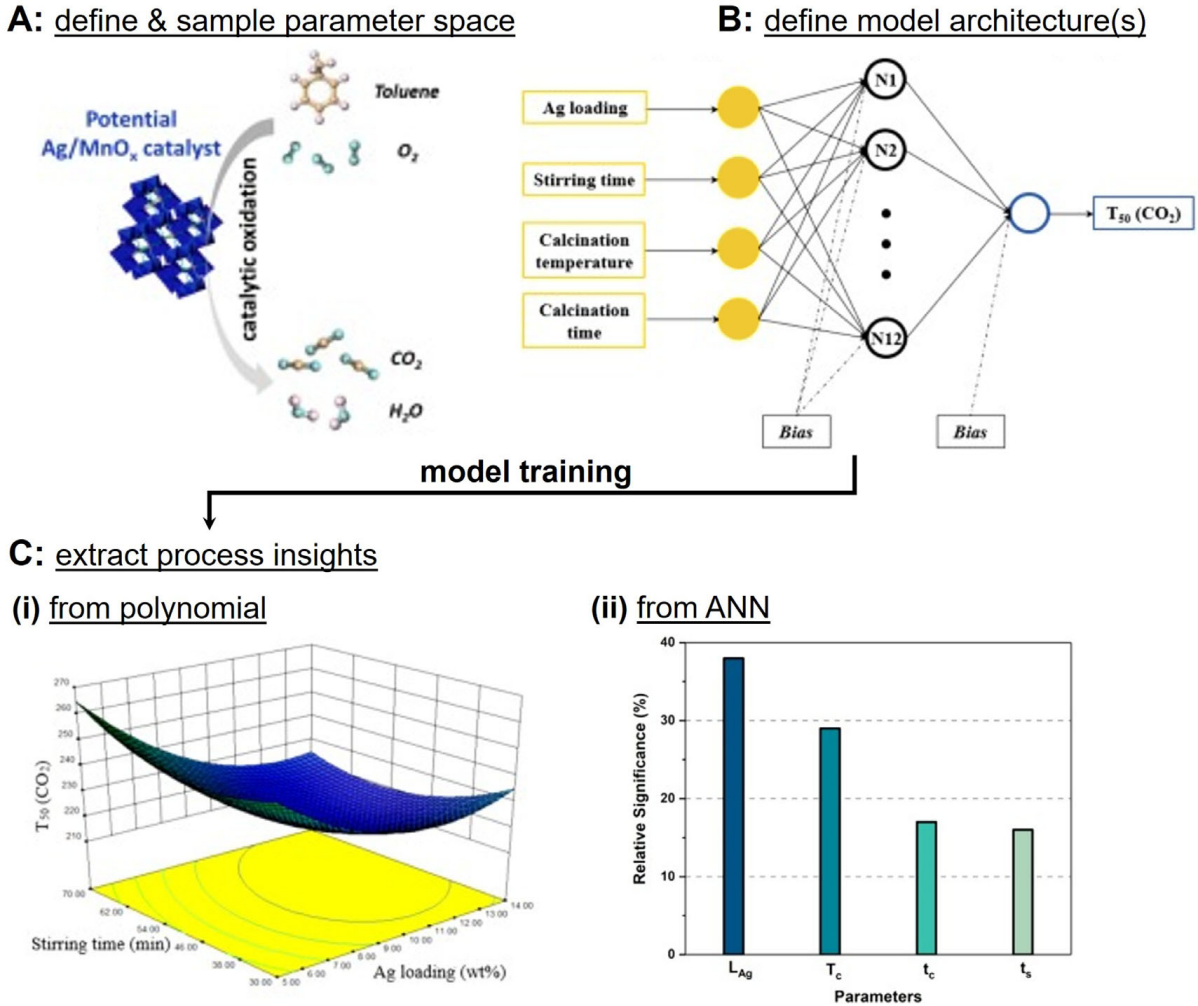
Guide for the inverse design of Ag/MnO_x_ catalysts with targeted catalytic performance, via a data‐based full property–process relationship, as outlined in Figure 2. (A) The relevant process parameters of the precipitation synthesis are identified and their ranges define the parameter space, which is systematically sampled using a central composite design. For each sampled condition, the catalytic performance of the resulting Ag/MnO_x_ catalyst is determined in the oxidation of toluene to carbon dioxide (T_50_(CO_2_)). (B) The sampled process parameters (input) and the corresponding catalytic performance (output) form the training dataset for the surrogate models. Two model architectures are trained on this dataset: a second‐order polynomial regression model and an artificial neural network (shown). (C) Process insights extracted from the trained models. The shape of the objective function landscape, shown here as a section through two of the four process parameters, is obtained from the polynomial model (i), while the relative significance of each process parameter for the catalytic activity is derived from the neural network (ii). Reprinted from Applied Catalysis A: General, Vol. 622, J. Yang, Z. Ye, G. Wang, L. Zhao, X. Zhang, J. Wang, and Y. Jin, “Neuro‐genetic machine learning framework accelerates the optimization of Ag/MnO_x_ catalyst for total oxidation of toluene”, 118221, Copyright (2021), with permission from Elsevier [217].

Two different surrogate models were then trained on this dataset (Figure 11B): a second‐order polynomial, analogous to the regression model discussed in Section 3.2.3.1, and a three‐layer artificial neural network (ANN) comprising four input neurons for the process parameters, twelve hidden neurons, and one output neuron for T_50_(CO_2_). The network was trained using leave‐one‐out cross‐validation to avoid overfitting and reaches *R* ^2^ value of 0.99 compared to 0.96 for the polynomial model. Comparing both data‐based models thus reveals that the more flexible function class (ANN) captures the shape of the property–process relationship more accurately, while the polynomial model retains a higher interpretability.

With the two trained property–process relationships at hand, three types of process insights were extracted (Figure 11C): (1) the process conditions yielding the highest catalytic activity, i.e., the lowest T_50_(CO_2_), which refers to the initial goal of inverse design; (2) the shape of the objective function landscape, which reveals whether the optimum is robust and whether alternative optima exist (see Section 1); and (3) the relative significance of each process parameter. For the ANN, the optimum was located using a GA, converging toward a silver loading of 13.2 wt%, a stirring time of 41.5 min, a calcination temperature of 461°C, and a calcination time of 4.3 h. Numerical optimization of the polynomial response surface returned an optimum at 11.9 wt%, 52.7 min, 410.9°C, and 4.1 h. The shape of the objective function landscape was resolved for the polynomial model by evaluating the fitted function on a sufficiently fine grid (Figure 11C‐i). The resulting surfaces are smooth and exhibit a single, broad minimum, with each parameter showing a volcano‐shaped dependence around an interior optimum. Similar to the example of optical properties in the previous section, such a smooth landscape is efficient for data‐based models, as a small number of well‐placed sampling points suffices to locate the optimum reliably (see also Section 4). From the weights of the ANN, in turn, the relative significance of each process parameter was extracted (Figure 11C‐ii), identifying the silver loading as the dominant factor with 38% of the total influence, followed by the calcination temperature, while stirring time and calcination time play a subordinate role. That the landscape was resolved from the polynomial model and the parameter significance from the ANN reflects the authors' choice rather than a limitation of either model class, as both analyses are in principle accessible from either.

This case study demonstrates that the choice of target property does not alter the methodology. The same three‐step workflow of sampling, surrogate modeling, and optimization applies to catalytic activity as it does to color.

## 4 Summary and Outlook

With this perspective, we aim to provide a practical framework for implementing inverse design in the development of nanoparticulate materials, encompassing both individual NPs in suspension and particle assemblies. Here, we focus exclusively on synthesis processes and how they can be optimized with respect to particle size and size distribution. However, it should be noted that post‐synthetic refinement, such as chromatographic techniques [71, 230, 231, 232], are important steps in the process chain and can substantially narrow the size distributions. The optimization over multiple unit operations across the full process chain, however, is still in its infancy.

### 4.1 The Advantage of Inverse Design

A key insight that applies universally to every model presented in this perspective, regardless of whether it is knowledge‐based or data‐based, and regardless of whether it serves as a process or property function, is that any calibrated model inherently enables answers to the four design questions raised in the *Introduction* as fundamental limitations of the traditional trial‐and‐error approach. That is, whether the optimal structure has been identified (Q1), how robust this structure is against synthesis variations (Q2), whether a structure providing the desired property exists within the chosen material system or synthesis route at all (Q3), and whether the synthesis route enables applications beyond the initial design goal (Q4). All four can be addressed through inspection of the objective function landscape resulting from the respective model. A global optimum reveals whether the optimal structure or process has been identified (Q1), the gradient structure of the objective function landscape around this optimum quantifies the sensitivity to synthesis variations and thus the robustness of the solution (Q2), forward evaluation of the model across the parameter space establishes whether the target property is attainable within the chosen material system or synthesis route at all (Q3), and a systematic survey of the accessible property space reveals which further applications the route may support (Q4).

The identification of the true optimum (Q1) is most evident in the knowledge‐based design of silver–gold alloy NPs (Section 3.1.3), where a derivative‐based optimizer navigates an irregular objective function landscape to locate the global optimum (Figure 7D‐viii). The contrast between this minimum and the surrounding local minima explicitly shows whether the optimal solution has been found. Robustness (Q2) is most clearly addressed in the data‐based design of RHAs (Section 3.2.2), where the permutation importance analysis directly quantifies how sensitive the circular dichroism response is to geometry variations imposed by the fabrication process. Whether a property target is attainable within a given route at all (Q3) is specifically answered in the knowledge‐based design of magnetic nanodiscs (Section 3.1.2.2). There, the three accessible magnetic configurations are mapped as a function of nanodisc shape, delineating the shape regions in which each configuration occurs (Figure 5A). Finally, the capacity to uncover opportunities beyond the obvious (Q4) is best illustrated by the structural optimization of silica core–gold shell particles (Section 3.1.2.1), which was in fact the starting point for this work. Mapping the complete accessible color space across core diameters and shell thicknesses (Figure 4A‐i) by simulation prior to any syntheses, identified colors that would have been difficult to locate experimentally and directed the synthetic effort toward predicted target structures from the beginning.

Taken together, these examples demonstrate that the transition from trial‐and‐error to inverse design is not merely a matter of convenience, it fundamentally reduces experimental effort and removes a substantial part of uncertainty from the optimization of nanoparticulate materials.

### 4.2 Knowledge‐ Versus Data‐Based Approaches

Building on the theoretical concepts of process and property functions, in this perspective we demonstrate how knowledge‐based and data‐based modeling approaches can be integrated with experimental data to construct models capable of optimizing synthesis conditions, nanoparticle structures, and resulting macroscopic properties. Both implementation strategies of inverse design offer significant opportunities but also exhibit inherent challenges and limitations. Ultimately, we believe that the goal of any model‐guided approach should be to advance understanding of the underlying physical and chemical processes and to uncover unifying principles governing nanoparticle formation and behavior. The knowledge‐based modeling approach is particularly valuable in this regard. Its implementation requires a substantial initial effort to gather detailed information on particle formation mechanisms and the relevant underlying physical and chemical phenomena. Moreover, it relies on access to key physical parameters, such as temperature and concentration profiles determined by reaction and mixing kinetics, as well as solubility data required to calculate supersaturation profiles. The availability of such data can be a limiting factor, particularly for novel components for which these parameters have not yet been established. In addition, the overall process must be decomposable into distinct steps, and the associated reaction network must be reducible to a manageable number of individual processes that accurately describe the reaction. In this context, post‐synthetic steps, or more generally, processes that are not explicitly captured by the model, must be negligible. This includes, for example, particle agglomeration in dispersion, implying that individual particles must exhibit sufficient colloidal stability. Knowledge‐based approaches thus often fail when applied to large, multicomponent systems where complexity is not manageable anymore. A calibrated mechanistic model, however, effectively serves as a digital twin of the physical system. When such a digital twin is employed as a process function, it not only enables the inverse design of NPs by linking process parameters to final particle characteristics but also facilitates time‐dependent process control, in which process parameters such as temperature or concentration can be dynamically adjusted throughout the process. While it is theoretically possible to capture such dynamic behavior using data‐based models, this is practically challenging because the data requirements scale exponentially with the required resolution in the discretization of the process control with regards to time and the need to establish input–output relationships at each time step. Furthermore, knowledge‐based models enable the extraction of additional information beyond the experimentally measured data, including transient or experimentally inaccessible quantities. Finally, once established, knowledge‐based approaches can often be transferred to related synthesis protocols as long as they are described by similar physicochemical principles. For example, many nanoparticle syntheses follow a classical reaction–nucleation–growth sequence. Once this relationship is mathematically established for one system, it can be extended to others by introducing additional mechanistic terms rather than developing an entirely new model. While this approach promotes a deeper understanding of nanoparticulate processes and can lead to the rational design of improved materials, those opportunities must be balanced against the entailing computational and experimental effort and the fact that many processes lack the availability of a reliable physical description.

In contrast, data‐based models enable inverse design without explicit knowledge of the underlying physics and chemistry. While in such models the mechanistic pathway remains unknown, they often yield comparable outcomes. This allows for rapid optimization and makes data‐driven approaches particularly attractive in productivity‐oriented or industrial environments. They are also indispensable in cases where few reliable physical models exist, for instance, in predicting catalytic or biomedical performance, which are often too complex to be described by rigorous models. While data‐based models primarily extract insights within the boundaries of the available dataset, that is, by interpolation and rarely by extrapolation, the trends and correlations they reveal still offer valuable mechanistic clues. Their performance, however, depends critically on the quantity and quality of their training dataset. Historically, the scarcity of large datasets has limited their application in materials science [157], but recent advances are helping to overcome this barrier. These include the development of algorithms capable of learning from smaller datasets [38, 76], as well as the growing use of automated synthesis platforms, such as robotic systems [112, 113] and continuous‐flow reactors [109, 233, 234, 235], which accelerate data generation via automation.

From a mathematical perspective, the decision to apply a knowledge‐based or a data‐based model to a given optimization problem can be reduced to three key factors: (i) experimental effort, (ii) numerical effort, and (iii) the shape of the objective function landscape linking a target property to a required structure or process parameters. Assuming the underlying physicochemical relationships of a system are well understood, knowledge‐based models typically require lower experimental effort but higher numerical effort. This is because limited experimental data are needed for model calibration, whereas identifying unique parameter combinations often necessitates extensive numerical exploration of the parameter space on fine grids to locate global optima. Conversely, data‐based models generally demand a high experimental effort due to the need for large training datasets, but require less numerical effort for both training and using the model. When considering the shape of the objective function landscape, important distinctions emerge. For nanoparticulate systems characterized by smooth objective functions, data‐based models perform well even when fitted to relatively few sampling points. In contrast, objective functions featuring narrow optima, multiple local minima, or elongated valleys require dense sampling to be accurately captured by data‐based approaches, often still resulting in limited model quality.

In simple terms, when focusing solely on predictive capability, a highly complex process that produces a smooth objective function can be efficiently modeled using data‐based approaches, for example employing low‐order polynomial regression functions requiring comparatively few data points. Conversely, even a simple process that yields a complex objective function landscape is better approximated using a knowledge‐based model.

This distinction can be illustrated by two examples from this perspective. In Section 3.1.2.1, we address the structural optimization of silica core–gold shell particles and demonstrate that core–shell Mie simulations provide a low‐complexity mechanistic description of the system. At the same time, the corresponding objective function landscape is highly complex, exhibiting multiple local optima and long, narrow valleys (Figure 4A‐iii). In this case, a knowledge‐based model was employed to reliably map the objective function. In contrast, Section 3.2.3.1 addresses the optimization of the transmission color of bimetallic AgAu alloy NPs. While the underlying system is highly complex (Figure 7B), the resulting objective function is smooth and well described by a quadratic function (Figure 10C‐i). Accordingly, a data‐based model was sufficient to capture the relevant relationships using as few as 30–40 data points.

### 4.3 Hybrid Approaches

While the advantages and limitations of knowledge‐based and data‐based modeling approaches can be discussed separately, their combination may offer another promising route forward. Such hybrid or gray‐box frameworks, which have a history in process engineering [236, 237], exploit the available physical knowledge while replacing unknown components with data‐based elements. Several architecturally distinct hybrid strategies may be envisioned to be relevant in the context of nanomaterial design, including (i) sequential coupling, (ii) physics‐informed data‐based modeling, and (iii) residual correction modeling.

Sequential coupling refers to the combination of a knowledge‐based process function with a data‐based property function, or vice versa. Such an approach can balance efficient nanoparticulate material design with the pursuit of a fundamental understanding of the underlying system. It may also help isolate the part of the property–process relationship that requires deeper mechanistic investigation. Physics‐informed data‐based modeling, including the increasingly prominent field of physics‐informed machine learning (PIML), incorporates physical laws, such as governing partial differential equations or conservation relationships, into the training of the underlying data‐based model, typically as additional constraints in the loss function [238, 239]. Beyond applications in chemical engineering [240], PIML has recently been applied to the inverse design of optical nanomaterials [241, 242, 243] and to the characterization of NPs via liquid phase TEM [244]. Lastly, residual correction modeling places a data‐based model in parallel with an imperfect or incomplete mechanistic model and trains it on the systematic discrepancy between mechanistic predictions and experimental observations. The data‐based component can compensate for missing physical effects without discarding the physical structure that is already known. Compared with a purely data‐driven model, this approach may therefore require fewer data while substantially improving predictive accuracy [236, 237]. This benefit comes at a cost, however, as the fitted parameters can no longer be interpreted as physically meaningful, because the data‐based component may compensate for physical effects that are not explicitly represented in the mechanistic model.

### 4.4 Choosing the Right Modeling Strategy

Selecting the most suitable modeling strategy for a given inverse design problem can be guided by two complementary sets of considerations. The first concerns the information available for the system at hand, namely:

(i) The degree of physicochemical understanding.
(ii) The expected complexity of the objective function landscape.
(iii) The type and amount of accessible data.

Together, these factors determine which modeling strategies can be implemented. The second set of considerations concerns the intended purpose of the model, namely:

(iv) The ability to extrapolate beyond the parameter space used for training.
(v) The ability to extract physically meaningful process insights.
(vi) The ability to optimize the system with regard to a number of parameters.

These factors determine which strategy should be chosen for a given inverse design problem, that is, which type of model is capable of delivering the intended purpose. In practice, the decision can be approached from either side, based on what information is available or from which model capability is required. Figure 12 presents qualitative model selection maps from the perspective of the available information, providing an intuitive picture on which modeling strategies are feasible for a given system, that is, which model can be constructed given a favorable value of the third dimension.

**FIGURE 12:**
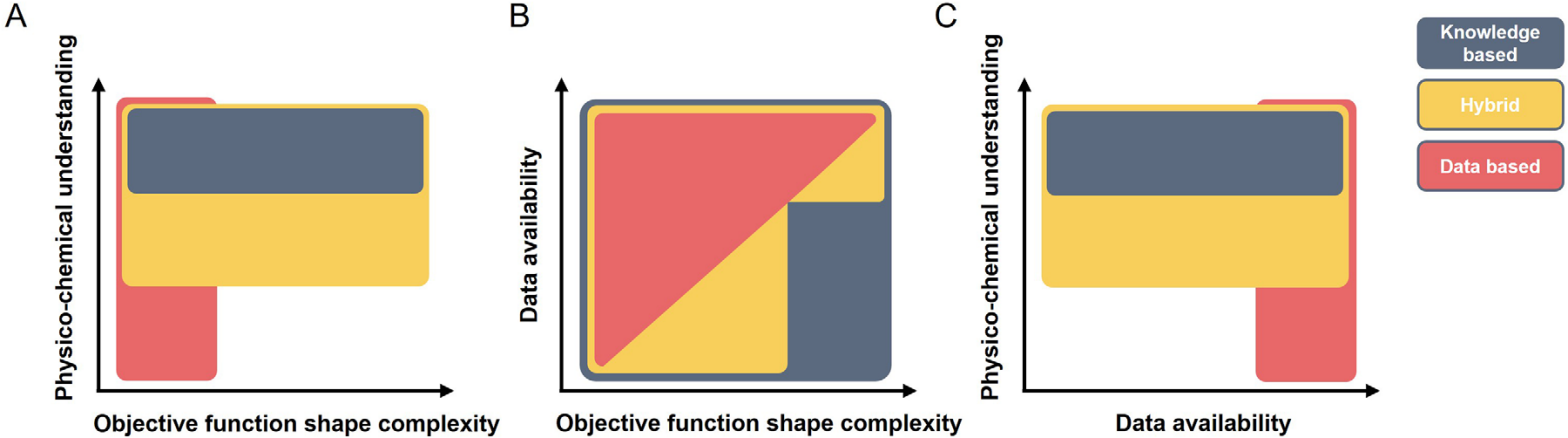
Qualitative model selection maps indicating which modeling strategy, that is, knowledge‐based (blue), hybrid (yellow), or data‐based (red), can be implemented for a given inverse design problem, based on three decision criteria, namely (i) the degree of physicochemical understanding, (ii) the complexity of the objective function landscape, and (iii) data availability. Since these criteria are interdependent, their pairwise relationships are shown in (A) physicochemical understanding versus objective function shape complexity, (B) data availability versus objective function shape complexity, and (C) physicochemical understanding versus data availability. Overlapping regions indicate that multiple strategies are simultaneously viable.

Figure 12A maps physicochemical understanding against objective function shape complexity. High physicochemical understanding enables knowledge‐based modeling across all levels of objective function complexity, while data‐based or hybrid models remain viable in parallel, provided sufficient data are available. When physicochemical understanding is limited, the appropriate strategy depends on the objective function structure. Smooth, low‐complexity landscapes are well captured by data‐based models even without mechanistic insight, while increasing complexity, for example, via narrow optima, multiple local minima, or elongated valleys, demands ever larger datasets that quickly become unavailable. Hybrid models, which incorporate available physical knowledge to constrain a data‐based surrogate, offer a practical compromise in this intermediate regime. When physicochemical understanding is low, but the objective function complexity is high, no reliable model can be constructed, and it becomes indispensable to deepen the mechanistic understanding of the system before attempting model development. Figure 12B maps data availability against objective function shape complexity. Because knowledge‐based models are grounded in physical principles rather than empirical correlations, their applicability is largely independent of the amount of available data given a sufficient physicochemical understanding of the system (blue region). Data‐based models, by contrast, require increasingly large datasets as objective function complexity grows (red region). Hybrid models bridge the gap at moderate complexity and limited data availability (yellow region), but below a critical data threshold and above a critical complexity, even hybrid approaches fail and a knowledge‐based description becomes necessary. Lastly, Figure 12C shows physicochemical understanding against data availability. High understanding supports knowledge‐based modeling largely regardless of the amount of available data. Abundant data enables data‐based modeling regardless of mechanistic insight. Intermediate combinations favor hybrid strategies. In the limiting case of simultaneously scarce data and low understanding, no strategy is reliably implementable.

Approaching model selection from the perspective of intended model capability, Figure 13 shows qualitative model selection maps indicating which modeling strategy is best suited to deliver each of the three key model outputs discussed above: The ability to extrapolate, extract meaningful process insights, or optimize the system with respect to a given number of parameters. The last criterion in particular refers not only to static optimization over a small set of parameters but also dynamic process optimization, for example, the determination of an optimal temperature profile over the course of a synthesis process. Such dynamic control is equivalent to optimizing process conditions at each time point throughout the process and thus represents optimization with respect to a large number of parameters.

**FIGURE 13:**
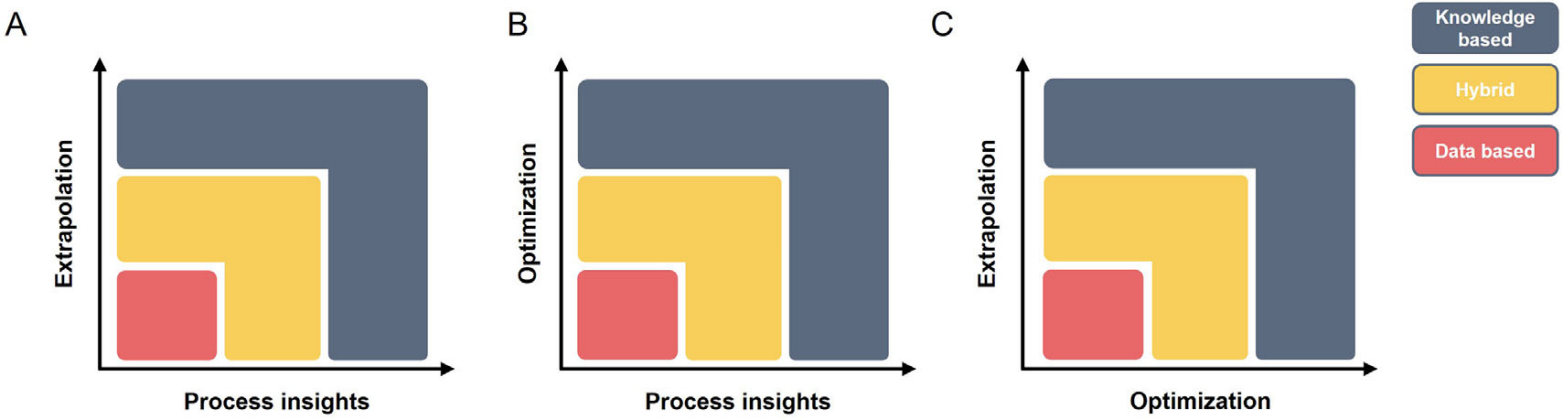
Qualitative model selection maps indicating which modeling strategy, that is, knowledge‐based (blue), hybrid (yellow), or data‐based (red), should be implemented for a given inverse design problem, based on three required model capabilities, namely (i) the ability to extrapolate beyond the parameter space used for training, (ii) the ability to extract physically meaningful process insights, and (iii) the ability to optimize the system with regard to a large number of parameters. Since these criteria are interdependent, their pairwise relationships are shown in (A) extrapolation versus process insights, (B) optimization versus process insights, and (C) extrapolation versus optimization.

Figure 13A maps the ability to extrapolate beyond the parameter space used for model training/calibration against the ability of the model to extract meaningful process insights. Data‐based models are well suited when neither significant extrapolation beyond the training parameter space nor mechanistic process insights are required. Knowledge‐based models, by contrast, are necessary when either high extrapolation capability or meaningful process insights are desired, as their physical grounding enables reliable predictions outside the calibration domain and provides interpretable parameters. Hybrid models occupy the intermediate regime, offering moderate extrapolation and partial process insight. Figure 13B maps optimization capability against process insight extraction. As above, extracting meaningful process insights requires a knowledge‐based or hybrid model. With respect to optimization, a key distinction arises between static and dynamic problems. Data‐based models are highly efficient for optimizing a small number of parameters in static settings. However, their data requirements grow rapidly with the number of optimization parameters, quickly exceeding the dataset sizes that are practically accessible in nanoparticle research. Dynamic process optimization, in which process conditions such as temperature or precursor concentration are adjusted continuously throughout a synthesis, is equivalent to optimizing over a large number of parameters (one per chosen time point) and therefore demands for a knowledge‐based model. Hybrid models again occupy an intermediate position which deliver partial process insights and are capable to optimize with regard to a moderate number of parameters while generating a moderate amount of process insights. Finally, Figure 13C maps extrapolation capability against optimization capability, that is, with regard to how many parameters the type of model can optimize a given system. It conveys a similar message where knowledge‐based models are required for far‐reaching extrapolation and high‐dimensional optimization, data‐based models are well suited for limited extrapolation and few optimization parameters, and hybrid models bridge the two extremes.

Taken together, Figure 12 and Figure 13 provide a practical decision framework that can guide the reader in selecting the most appropriate strategy for their specific inverse design problem. It should be noted that the choice of modeling strategy for any given inverse design problem is inherently case‐dependent and rarely permits a definitive answer. The selection criteria and maps presented above are therefore intended as qualitative decision aids rather than prescriptive rules, providing a structured starting point for identifying the most relevant considerations for a given system.

Independent of the preferred modeling strategy, with this perspective we aim to provide a practical starting point to adopt inverse design strategies and thereby accelerate the shift from empirical trial‐and‐error approaches toward predictive, inverse, and model‐guided design of nanoparticulate materials.

## Funding

We gratefully acknowledge the funding by the Deutsche Forschungsgemeinschaft (DFG, German Research Foundation)—Project‐ID 416229255 – SFB 1411.

## Conflicts of Interest

The authors declare no conflicts of interest.

## Data Availability

No substantially new data were generated or analyzed in this study. Data underlying the cited works are available as described in the respective publications.
